# Visualizing Cathepsin K‐Cre Expression at the Single‐Cell Level with GFP Reporters

**DOI:** 10.1002/jbm4.10706

**Published:** 2022-12-21

**Authors:** Wenhuan Chai, Weiwei Hao, Jintao Liu, Zhenglin Han, Shiyu Chang, Liben Cheng, Mingxin Sun, Guofang Yan, Zemin Liu, Yin Liu, Guodong Zhang, Li Xing, Hongqian Chen, Peng Liu

**Affiliations:** ^1^ Laboratory of Bone & Adipose Biology Shanxi Medical University Taiyuan China

**Keywords:** AGING, CATHEPSIN K, CRE, GFPS, OSTEOBLASTS, OSTEOCLASTS

## Abstract

The Cre/lox system is a fundamental tool for functional genomic studies, and a number of Cre lines have been generated to target genes of interest spatially and temporally in defined cells or tissues; this approach has greatly expanded our knowledge of gene functions. However, the limitations of this system have recently been recognized, and we must address the challenge of so‐called nonspecific/off‐target effects when a Cre line is utilized to investigate a gene of interest. For example, cathepsin K (Ctsk) has been used as a specific osteoclast marker, and Cre driven by its promoter is widely utilized for osteoclast investigations. However, Ctsk‐Cre expression has recently been identified in other cell types, such as osteocytes, periosteal stem cells, and tenocytes. To better understand Ctsk‐Cre expression and ensure appropriate use of this Cre line, we performed a comprehensive analysis of Ctsk‐Cre expression at the single‐cell level in major organs and tissues using two green fluorescent protein (GFP) reporters (ROSA nT‐nG and ROSA tdT) and a tissue clearing technique in young and aging mice. The expression profile was further verified by immunofluorescence staining and droplet digital RT‐PCR. The results demonstrate that Ctsk‐Cre is expressed not only in osteoclasts but also at various levels in osteoblast lineage cells and other major organs/tissues, particularly in the brain, kidney, pancreas, and blood vessels. Furthermore, Ctsk‐Cre expression increases markedly in the bone marrow, skeletal muscle, and intervertebral discs in aging mice. These data will be valuable for accurately interpreting data obtained from in vivo studies using Ctsk‐Cre mice to avoid potentially misleading conclusions. © 2022 The Authors. *JBMR Plus* published by Wiley Periodicals LLC on behalf of American Society for Bone and Mineral Research.

## Graphical abstract

We performed a comprehensive analysis of Ctsk‐Cre expression at the single‐cell level in major organs and tissues using two GFP reporters. These results demonstrate that Ctsk‐Cre is expressed not only in osteoclasts but also at various levels in osteoblast lineage cells and other major organs/tissues, particularly in the brain, kidney, pancreas, and blood vessels. These data will be valuable for accurately interpreting data to avoid potentially misleading conclusions.

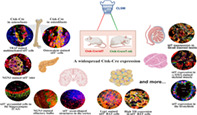

## Introduction

The development of the Cre/lox system for conditional gene targeting, which has allowed spatial and temporal localization of genetic manipulation, has expanded and refined the genetic manipulation toolbox.^(^ ^1^ ^)^ In recent decades, this system has provided a powerful tool to substantially boost our knowledge of bone biology. However, the limitations of this system have been gradually acknowledged, as mentioned in several excellent reviews^(^ ^2^, ^3^, ^4^, ^5^ ^)^ emphasizing that caution must be used when drawing conclusions from experiments in which a Cre line is used because of the “nonspecific” expression observed in many generated Cre lines; this expression does not follow the documented expression pattern for which the selected promoter was originally characterized. Nonspecific expression can lead to misinterpretation of phenotypes, so the tissue or cell specificity of Cre expression should be evaluated carefully. Reporter mouse lines are needed to resolve complexity and provide an accurate readout of Cre activity.

Several osteoclastic Cre lines have been developed to investigate osteoclasts, including lines using tartrate‐resistant acid phosphatase (TRACP) and cathepsin K (Ctsk) promoter drivers.^(^ ^6^, ^7^, ^8^ ^)^ Ctsk is a cysteine protease that is significantly upregulated during osteoclastogenesis,^(^ ^9^, ^10^ ^)^ and CTSK deficiency is known to cause pycnodysostosis or Toulouse‐Lautrec syndrome, a rare skeletal dysplasia characterized by bone abnormalities, such as short stature, acroosteolysis of the distal phalanges, and skull deformities.^(^ ^11^, ^12^, ^13^, ^14^ ^)^ Ctsk is widely used as an osteoclast‐specific marker to guide Cre activity.^(^ ^6^, ^7^, ^8^ ^)^ However, its expression has been recently observed in osteocytes,^(^ ^15^, ^16^ ^)^ skeletal stem cells,^(^ ^17^ ^)^ and tendon cells,^(^ ^18^ ^)^ as well as other cell types, as shown at the mRNA level in public databases (e.g., BioGPS, FANTOM 5, and Tabula Muris) and other works.^(^ ^19^, ^20^, ^21^ ^)^ However, the original description of Ctsk‐Cre^(^ ^22^ ^)^ and a recent work^(^ ^23^ ^)^ claimed that it is specific to osteoclasts, without any detectable expression in osteoblasts or other examined tissues, such as the growth plate, cartilage, brain, hypothalamus, testis, ovary, pancreas, liver, small intestine, kidney, and thyroid. To address this dispute and thoroughly evaluate the Ctsk‐Cre expression profile at the single‐cell level, we systemically assessed its expression patterns with two green fluorescent protein (GFP) reporters in young and aging mice, as the expression patterns of different reporters may vary due to the different structures of the reporters. It is expected that a better understanding of Ctsk‐Cre expression will be valuable for improving the utilization of this Cre tool. A tissue clearing technique was also employed in this study, as it is known to improve sensitivity for detecting fluorescence signals and reduce the baseline level of background fluorescence.^(^ ^24^ ^)^

## Materials and Methods

### Mice

Cathepsin K‐Cre mice (Ctsk‐Cre) were provided by A. Qin and were originally from S. Kato^(^ ^22^ ^)^; C57BL/6‐Gt(ROSA)26 or tm5(CAG‐LSL‐tdTomato)/Bcgen (tdTomato cKI) mice were purchased from Biocytogen (Biocytogen, Beijing; Stock No. 110148); and B6;129S6‐Gt(ROSA)26Sor^tm1(CAG‐tdTomato*,‐EGFP*)Ees^/J (ROSAnT‐nG) mice were purchased from JAX (Stock No. 023035). All mice were maintained on a C57BL6/J background throughout the study. All animals were housed in a pathogen‐free environment in a vivarium approved by the Association for Assessment and Accreditation of Laboratory Animal Care (AAALAC) of Shanxi Medical University; experiments were performed in accordance with the guidelines of the Institutional Animal Care and Use Committee (IACUC).

The following breeding strategy was used to produce Ctsk‐Cre^+^:tdT/nT‐nG reporter mice. Breeding pairs were established in their own cages by mating a 2‐ to 3‐month‐old male carrying the Ctsk‐Cre^Tg/+^ allele with two or three 2‐ to 3‐month‐old females carrying the tdT^flox/flox^ or nT‐nG^flox/flox^ allele. The offspring were then weaned at 3–4 weeks after birth and separated into different cages by sex. After genotyping, the male littermates harboring the Ctsk‐Cre ^Tg/+^:tdT^flox/+^ or nT‐nT^flox/+^ allele were used for analysis, while age‐matched pure tdT ^flox/flox^ or the nT‐nT^flox/flox^ mice from each maintaining cage were used as the negative controls instead of Ctsk‐Cre littermates. The ages of the tested mice are indicated for each analysis.

The primers used for genotyping are as follows: (i) for Cre: Fwd 5′ ACTCCTTCATAAAGCCCT 3′ and Rev 5′ ATCACTCGTTGCATCGACCG 3′; (ii) for authentication of the Ctsk‐Cre line: FCtsk 5′ CCATTCGTGTCTGAGGCTTT 3′ and RCre 5′ CATCGACCGGTAATGCAG 3′^(^ ^25^ ^)^; (iii) for Rosa nT‐nG: mutant Fwd 5′ CCAGGCGGGCCATTTACCGTAAG 3′, common Fwd 5′ AAAGTCGCTCTGAGTTGTTAT 3′, and wild type 5′ GGAGCGGGAGAAATGGATATG 3′; and (iv) for Rosa‐tdT: ROSA‐GT‐F 5′ AGTCGCTCTGAGTTGTTATCAG 3′, ROSA‐GT‐R 5′ TGAGCATGTCTTTAATCTACCTCGATG 3′, and mutant ROSA‐GT‐F 5′ AGTCGCTCTGAGTTGTTATCAG 3′.

### Cryosectioning

Cryosectioning was performed as described previously.^(^ ^26^ ^)^ Briefly, freshly harvested samples were fixed with 4% paraformaldehyde (PFA) (RH168295, Rhawatm, Shanghai, China) for 12–24 h at 4°C. For hard tissues, the samples were washed with 1X PBS and decalcified with 0.5 M EDTA (F191352, Aladdin Biochemical Technology, Shanghai, China) for 7 days. The solution was changed daily. Then they were transferred to 30% sucrose in 1× PBS overnight. The tissues were embedded with optimal cutting temperature compound (OCT) stored at −80°C. These samples were sectioned into 7‐ to 45‐μm sections using a Leica cryostat CM1950.

### Tissue clearing

The PEGASOS method was used for tissue clearing, as described previously.^(^ ^27^ ^)^ In short, the mice were deeply anesthetized and transcardially perfused with 0.1 M 1× PBS, followed by 4% PFA. Tissues were removed and postfixed overnight in 4% PFA. The skeletal tissues were decalcified with 0.5 M EDTA at 4°C for 3–5 days to improve the clearing effect. The EDTA solution was replaced daily with fresh solution. In the last phase of demineralization, the skeletal tissues were sectioned with a Leica cryostat to thin them to further improve the clearing effect. Then the decalcified bones and soft tissues were soaked in primary clearing buffer containing 40% tert‐butyl alcohol, 30% PBS, 20% Triton TMX‐100 (SLBZ8156, Sigma), and 10% ethanolamine (SHBG0568 V, Sigma) at 4°C for 3 days, and the buffer was replaced every day. The samples were immersed in secondary buffer with 50% tert‐butyl alcohol, 20% PBS, 20% Triton‐100, and 10% ethanolamine at 4°C for 2 days, which was replaced with fresh buffer daily. Finally, the samples were placed in the final clearing solution buffer of 70% tert‐butyl alcohol, 10% PBS, 20% Triton‐100, and 10% ethanolamine at 4°C for 1 day. These prepared tissues were then subjected to imaging with a Nikon A1 HD25 confocal microscope.

### Immunofluorescence staining

Immunofluorescence staining was performed according to the manufacturers' recommendations. Briefly, air‐dried 7‐ to 45‐μm‐thick frozen sections were washed with 1× PBS for 10 minutes three times, followed by blocking with 10% BSA for 20 minutes at room temperature. If needed, the samples were permeabilized with 0.5% Triton X‐100 in 1× PBS for 10 minutes. Then diluted primary antibodies were added to the slides. After overnight incubation with antibodies at 4°C, the slides were washed with 1× PBS for 15 minutes three times and then incubated with secondary antibody conjugated with fluorescence for 30 minutes at room temperature, followed by washing again thoroughly with 1× PBS for 10 minutes three times. The slides were then stained with DAPI, rinsed with 1× PBS for 5 minutes three times, and mounted with 50% glycerol or fluoremount‐G for confocal imaging. Rabbit IgG was used as the negative control. Primary and secondary antibodies were purchased from Servicebio (Wuhan Servicebio Technology Co., Wuhan, China), except when indicated otherwise. The primary and secondary antibodies are listed in Table 1.

**Table 1:** List of Antibodies Used for Tissue/Cell Identification

Primary antibodies [rabbit anti‐mouse (IgG) abs]					Secondary antibody
Name	Sources	Catalog No.	Dilutions	Tissues/cells	
NeuN	Servicebio	GB11138	1:500	Brain	Goat Anti‐rabbit IgG (H + L) (Alexa Fluor® 488 conjugate) Catalog No. GB25303 Dilution: 1:400. Color: green
IBA1	Servicebio	GB113502	1:500	Brain	
GFAP	Servicebio	GB11096	1:800	Brain	
F4/80	Servicebio	GB113373	1:800	Brain, kidney, liver, lung, pancreas, WAT, and BAT	
Neurogenin 3	Bioss	bs‐0922R	1:100	Brain	
			1:300	Pancreas	
ɑ‐SMA	Servicebio	GB111364	1:200	Heart	
			1:500	Muscle	
Type 1 collagen	Servicebio	GB11022‐3	1:800	Kidney and liver	
CD44	Servicebio	GB112054	1:300	Lung	
Stra8	Bioss	bs‐1903R	1:300	Testis	
Ucp1	Servicebio	GB112174	1:300	WAT and BAT	
TH	Servicebio	GB11181	1:500	WAT	
Calcitonin R	Bioss	bs‐0124R	1:400	Patella and femur	
Osteocalcin	Servicebio	GB11233	1:400	Tibia	
Tracp/CD40 L	Servicebio	GB11416	1:500	Tibia	
Osteopontin	Servicebio	GB11500	1:500	Tibia	
Sp7	Servicebio	GB111900	1:300	Calvaria	
CD31	Servicebio	GB11063‐2	1:100	Liver, muscle, and tibia	
EMCN	Santa Cruz	sc‐65495	1:100	Tibia	

### Imaging

Frozen/tissue‐cleared sections were washed three times with 1× PBS for 5 minutes each time. DAPI (0.5 mM) was added and incubated at room temperature for 1–2 minutes. The sections were mounted with 50% glycerin solution in 1× PBS. The fluorescence images of the frozen and tissue‐cleared sections were obtained using a Nikon A1 HD25 confocal microscope with a DUVB detector and plan Apo λ 4×, plan Apo VC 20× DIC N2, plan Apo λ 40×, and plan Apo λ 100×C oil objectives, illuminated with a wavelength of 405, 488, or 561 nm to excite DAPI, GFP, or tdTomato, respectively; detection was performed with a 425–475, 500–550, or 570–620 nm bandpass filter. To assess the number of cells in each field of view, tissue‐cleared images were converted from 3D to MAIP (maximum projection of the Z‐stack across the whole section). Data were acquired and analyzed using NIS‐Elements AR 5.20.00 64‐bit software.

### Culture of osteoclasts and osteoblasts

Mouse bone marrow‐derived stromal cell (mBMSC) cultures were conducted as described previously.^(^ ^28^ ^)^ For osteogenic differentiation, cultured mBMSCs were induced with osteogenic differentiation medium [α‐MEM containing ascorbic acid (50 μg/mL) and β‐glycerophosphate (10 mM) and dexamethasone (10^−8^ M)] for 20–25 days. The differentiation medium was then changed every 2 days. Osteoclast differentiation was performed as described previously.^(^ ^28^ ^)^ In brief, bone marrow cells were induced to differentiate into osteoclasts with receptor activator of NF‐κB ligand (RANKL) (25 ng/mL) and macrophage colony‐stimulating factor (M‐CSF) (30 ng/mL) (Sino Biological, Beijing). The medium was refreshed daily for 7 days. Osteoclast formation was then examined under a Nikon A1 HD25 confocal microscope.

### Total RNA extraction

Tissues were harvested from 10‐week‐old B6 mice. Samples were dissected free of connective tissue and minced with scissors, and total RNA was extracted using NucleoZOL (NucleoZOL; Macherey‐Nagel GmbH & Co., KG, Dylan, Germany). mRNA was reverse transcribed using an M5 Super qPCR RT kit with gDNA remover (MF012‐T, Mei5bio, Beijing).

### Droplet Digital™ PCR

Droplet digital PCR was performed according to the manufacturer's guidelines (Bio‐Rad QX200 ddPCR system, Bio‐Rad, USA) as described previously.^(^ ^29^ ^)^ The 20‐μL ddPCR system included the QX200 ddPCR EvaGreen® Supermix 200 reagent, 2.5 ng/μL cDNA, and 500 μM primer. For droplet generation, the 20‐μL reaction was transferred into the middle row of a DG8™ Cartridge, and 70 μL of droplet generation oil was added to the bottom wells of the DG8™ Cartridge for the QX200 Droplet Generator. After generating droplets, the emulsified samples were transferred from the top wells of the cartridge into a PCR plate (ddPCR TM96‐Well Plates, Bio‐Rad‐12001925) and sealed with the PX1TM PCR Plate Sealer. Then the plate was placed in a PCR thermal cycler to amplify the fluorescence intensity. The PCR procedure was designed to include a step of 95°C for 5 minutes, followed by 40 cycles of a three‐step cycling protocol (95°C for 30 seconds, 58.6°C for 60 seconds and 72°C for 30 seconds), and the last step was 95°C for 10 minutes. The ramp rate between steps was slowed to 2°C/s. The data were analyzed with QantaSoft Software (version 1,7,4). The primers used for dd‐RT–PCR were as follows: mCtsk Fwd‐5′ CAGTGTTGGTGGTGGGCTAT 3′; mCtsk Rev‐5′ CATGTTGGTAATGCCGCAGG 3′.

### Statistical analysis

All data are shown as the mean ± SEM. The differences between tested groups were analyzed by a two‐tailed, unpaired Student's *t*‐test using GraphPad Prism version 8.0.1. Differences were considered statistically significant at *p* < 0.05, *n* = 3.

## Results

To better assess Ctsk‐Cre reporter expression, we chose two reporter lines, tdT^flox/flox^ and nT‐nG^flox/flox^, as different promoter‐driven reporters have shown variable expression patterns.

In tdT mice, strong red fluorescence is expressed in whole cells when Cre is present. In contrast, baseline fluorescence is low in the absence of Cre, which is similar to Ai9.^(^ ^30^ ^)^

In dual‐color nT‐nG reporter mice, nucleus‐localized strong red fluorescence (nT) is expressed in a widespread fashion in the absence of Cre, with a switch to nucleus‐localized GFP (nGFP) expression in the presence of Cre.^(^ ^31^ ^)^ This dual‐color system allows direct visualization of both Cre recombined cells and nonrecombined cells at single‐cell resolution.

The examined organs and tissues were harvested from Ctsk‐Cre:tdT mice at 2 months of age and Ctsk‐Cre:nT‐nG mice at 1 or 8 months of age or when indicated. Authentication of the Ctsk‐Cre line was confirmed by promoter‐ and Cre‐specific genotyping PCR with a 992 bp amplicon (Data S1).^(^ ^25^ ^)^ These samples were processed by tissue clearing and/or frozen sectioning, followed by confocal imaging. Pure floxed mice were used as negative controls instead of littermates. Negative control samples were imaged under the same conditions used for the Cre^+^ samples. The results for nT‐nG and tdT are shown in Data S2.

### Ctsk‐Cre expression in skeletal system

#### In femur

**Fig. 1:**
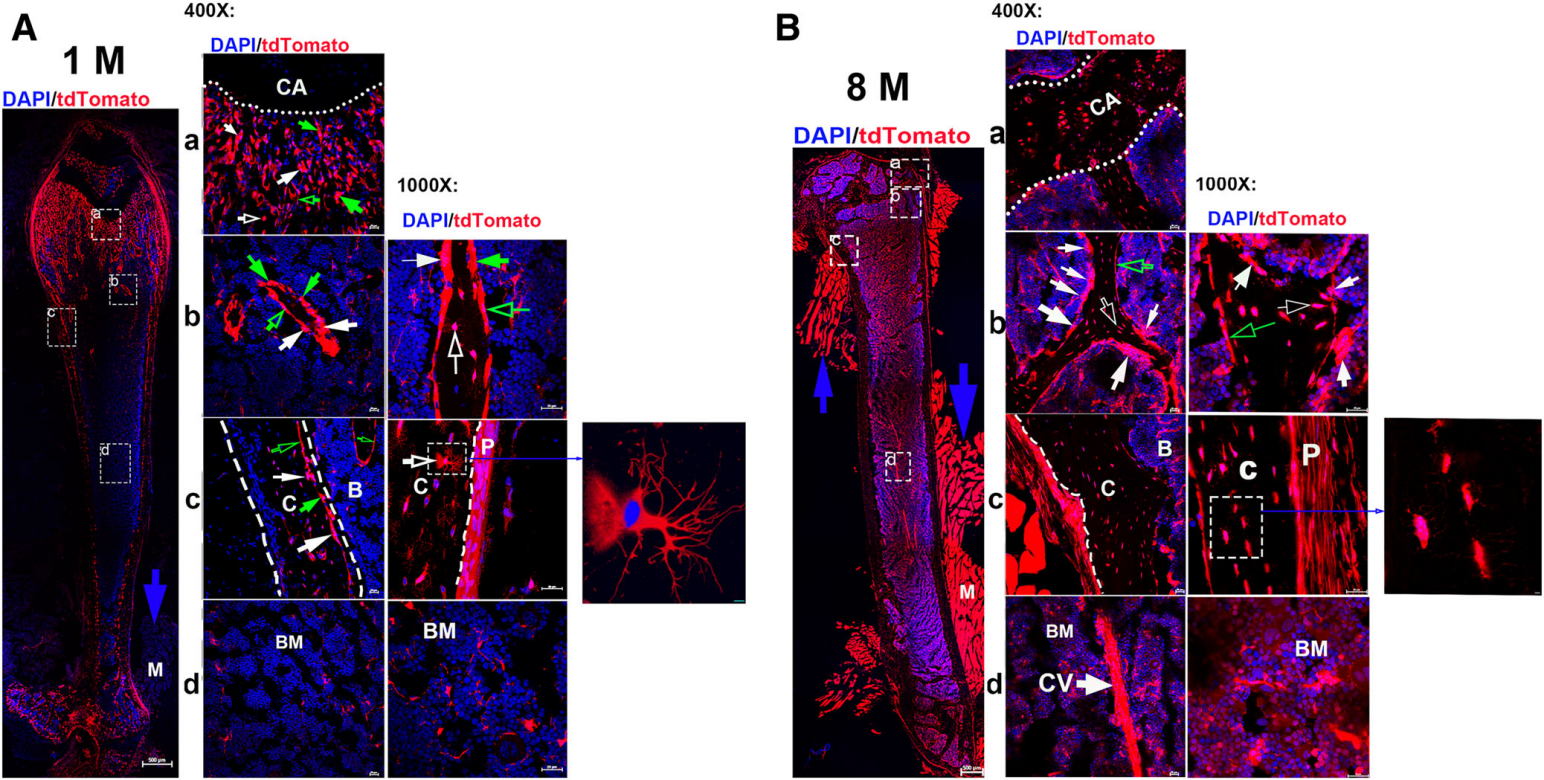
Visualization of Ctsk^+^ cells in Ctsk‐Cre^+^:tdT mice. Whole‐femur imaging was performed on frozen sections from mice aged 1 month (*A*) and 10 months (*B*), with representative areas shown at higher magnifications as indicated (×400 and ×1000). The representative areas include the growth plates (*a*), trabecular bones (*b*), cortical bones (*c*), and bone marrow (*d*), with enlarged images of osteocytes with dendrites. Arrows: white: tdT^+^ cells with more than two nuclei on the resorption bays of bone surfaces are osteoclasts; green: cuboid‐shaped tdT‐positive cells with a single nucleus on the bone surfaces are osteoblasts; empty white: osteocytes embedded within bone matrix; empty green: flat cells lining bone surface are bone lining cells. CA, cartilage; C, cortical bones; P, periosteum; BM, bone marrow. Scale bars, 500 μm for whole‐femur images with a magnification of ×40; 20 μm for representative areas at magnifications of ×400 and ×1000; and 10 μm for images of osteocytes.

We first examined the femora of young and aging Ctsk‐Cre^+^:tdT mice to capture images of tdT expression at different magnifications. In the young mice, high tdT expression was detected in spongy bone of the epiphysis, endosteal surface, and periosteum at low magnification (Fig. 1*A*, left panel). Then the representative areas were further examined at higher magnifications. The images showed tdT expression in cuboid and nucleated cells on the trabecular and endosteal surfaces (green arrows), such as osteoblasts, and multinucleated cells (more than two nuclei) on resorption bays of the trabecular and endosteal surfaces (white arrows), such as osteoclasts (Fig. 1*A‐a–c*). In addition, tdT was observed in bone lining cells and osteocytes (empty green and white arrows, respectively) (Fig. 1*A‐b,c*). In the bone marrow, tdT was observed in fibroblast‐like cells or vascular structures (b and c). tdT was also clearly expressed in the cellular dendrites of osteocytes (Fig. 1*A‐c*). Unexpectedly, however, it was not detectable in the articular cartilage and growth plate (Fig. 1*A‐a*).

In contrast, several obvious differences were observed during aging (Fig. 1*B*): (i) tdT^+^ cells appeared in the growth plate and were markedly increased in the bone marrow (Fig. 1*B* the right panel, and a and d); (ii) an expanded and loose tdT^+^ periosteal layer could be seen (Fig. 1*B‐c*); (iii) an increase in osteocyte numbers occurred, with loss of their dendritic structure (Fig. 1*B‐c*); (iv) an increase in multinucleated tdT^+^ cells was observed (Fig. 1*B‐b*); and (v) strong tdT expression was detected in skeletal muscle indicated by blue arrows (Fig. 1*B*, right panel).

#### In spine

**Fig. 2:**
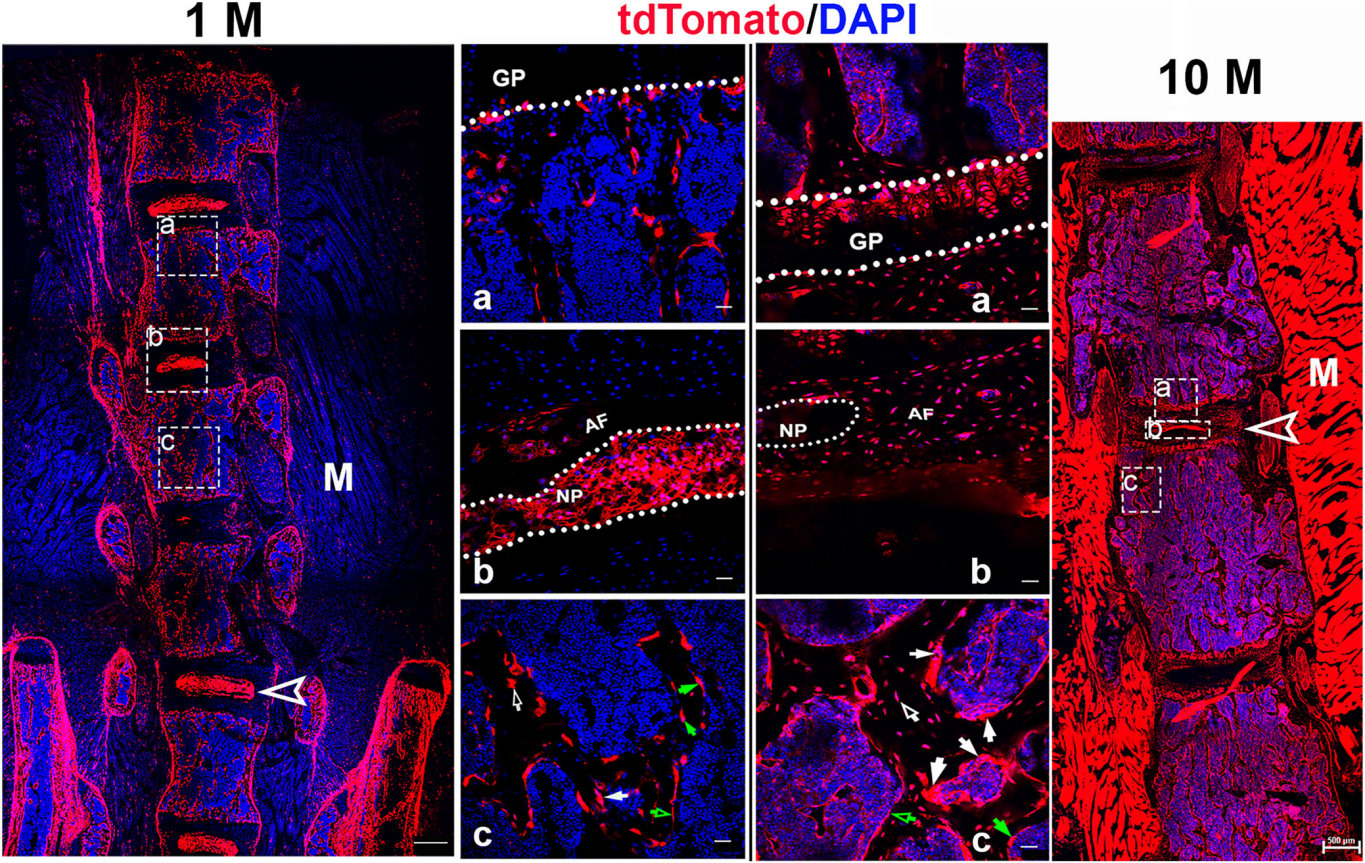
Capturing Ctsk expression in spine. Images were taken of spine sections from Ctsk‐Cre^+^:tdT mice aged 1 month and 10 months. The representative areas from each section are shown in the growth plate (*A*), IVD (*B*), and trabecular bone (*C*). Arrows: white: tdT‐positive cells with more than two nuclei on resorption bays of bone surfaces, osteoclasts; green: cuboid‐shaped tdT‐positive cells with a single nucleus on bone surfaces, osteoblasts; empty white: osteocytes embedded within bone matrix; empty green: flat cells lining bone surface, bone lining cells; and empty arrowheads: IVD. M, muscle; GP, grown plate; AF, annulus fibrosus; NP, nucleus pulposus. Scale bars, 500 μm for whole images of spines and 20 μm for magnified images of representative areas. Magnifications: ×40 for whole‐spine images; ×400 for representative images.

We further confirmed tdT expression in the spine. The expression profile was similar to that in the femur, except for tdT expression in the intervertebral disc (IVD) (Fig. 2). In the young mice, we observed a higher level of tdT expression in the nucleus pulposus (NP) than in the annulus fibrosus (AF) (Fig. 2*A*); however, in aging mice, an increase in tdT expression was found in the AF, and the NP was smaller and dislodged, retaining its tdT expression levels (Fig. 2*B*). The IVDs are indicated by empty white arrowheads in Fig. 2 (left panel: 1 M; right panel: 10 M).

#### Ctsk expression in Ctsk‐Cre ^+^:nT‐nG Mice

**Fig. 3:**
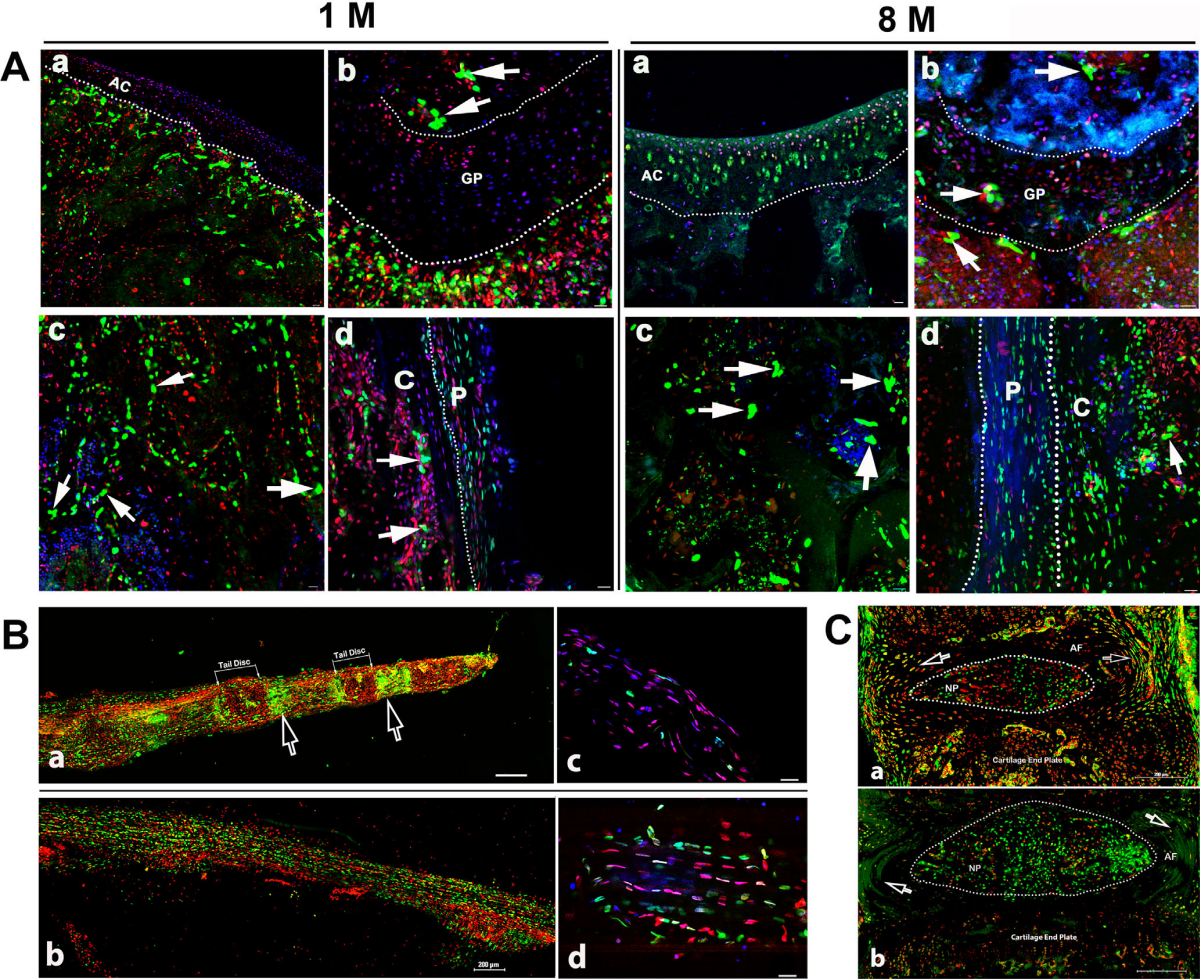
Confocal imaging of Ctsk^+^ cells in Ctsk‐Cre^+^:nT‐nG mice. (*A*) Documentation of Ctsk^+^ cells (nG^+^) in femoral sections. Imaging of Ctsk^+^ cells was performed for young (*A*, aged 1 month) and aging mice (*B*, aged 8 months). The representative areas are shown as follows: a: articular cartilage; b: growth plate; c: trabecular bones; and d: cortical bone close to growth plate. White arrows indicate multinucleated nGFP‐expressing cells (mnGFPs). AC, articular cartilage; GP, grown plate; C, cortical bones; P, periosteum. Scale bars, 20 μm. Magnification: ×400. (*B*) Imagining Ctsk^+^ cells in whole tails. Whole tails were imaged for mice aged 1 month (*a*) and 8 months (*b*). High‐magnification images of fibrous layer from each are shown in (*c, d*), respectively. White arrows indicate bright nG expression under growth plates. Scale bars, 200 μm for (*a*, *b*); 20 μm for (*c*, *d*). Magnification: ×200 for (*a*, *b*) and ×400 for (*c*, *d*). (*C*) Alteration of Ctsk expression in intervertebral discs (IVDs) with aging. The IVDs were imaged in 1‐month‐old mice (*a*) and 8‐month‐old mice (*b*). Dotted ellipses indicate NP area. Empty white arrows indicate area of AF. AF, annulus fibrosus; NP, nucleus pulposus. Scale bars: 200 μm. Magnification: ×200.

Taking advantage of two reporters simultaneously, we further investigated Ctsk expression in Ctsk‐Cre^+^:nT‐nG mice. We found two types of nGFP^+^ cells derived from nT: multinucleus nGFP‐expressing cells (mnGFPs) (white arrows) and single‐nucleus nGFP‐expressing cells (snGFPs) (Fig. 3*A*). The nG expression pattern was strongly analogous to that of tdT, as shown in Fig. 3*A*. In addition, we further confirmed Ctsk expression in articular cartilage and the growth plate in aging mice, but this pattern was not observed in young mice (Fig. 3*A‐a,b*).

Next, the whole tail was imaged for Ctsk expression after the tissue clearing process. nGFP expression was observed in the tendon and intervertebral disc; in particular, higher expression was seen under the growth plates in the young mice (white arrows) (Fig. 3*B‐a*), but this high nGFP expression disappeared in the aging mice (Fig. 3*B‐a*), which made it difficult to recognize tail discs. In addition, nG expression increased in the tendon during aging (Fig. 3*B‐d*) compared to the young tendon (Fig. 3*B‐c*) at high magnification.

Moreover, we specifically examined nGFP expression in the IVD at high magnification. nGFP expression was observed in both the AF and NP in the young mice; however, in the aging mice, this expression significantly increased in the NP (dotted ellipse) (Fig. 3*C‐a*), whereas it was substantially reduced in the AF (empty white arrows) (Fig. 3*C‐b*).

#### In sutures

**Fig. 4:**
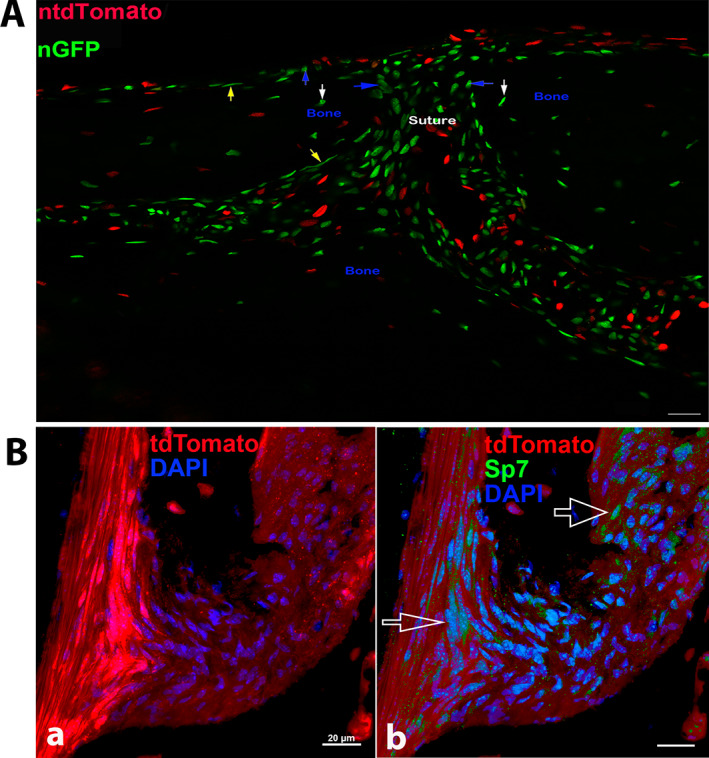
Representative images of Ctsk^+^ cells in sutures. Representative image of cranial suture of Ctsk‐Cre^+^:nT‐nG mice aged 1 month (*A*). Arrows: yellow: bone lining cells; blue: cuboidal nG+ cells on bone surface; white: nG^+^ cells embedded within bone matrix. Scale bars, 20 μm. Magnification: ×400. Representative images of sutures from Ctsk‐Cre^+^:tdT mice (*B*). tdT expression (*a*) and immunofluorescence staining with Sp7 antibody (*b*). Empty white arrows indicate Sp7‐positive cells. p, periosteum; s, suture. Scale bars, 20 μm. Magnification: ×1000.

After the sutures of Ctsk‐Cre^+^:nT‐nG mice were imaged, nGFP^+^ cells, such as osteocytes, were found in mineralized bone (white arrows) (Fig. 4*A*). In addition, nGFP was observed in cells on the cranial surface, such as osteoblasts or bone lining cells (blue and yellow arrows, respectively) (Fig. 4*A*). Furthermore, nGFP was also located in cells in the pericranium/periosteum and the sagittal suture mesenchyme between the parietal bones (Fig. 4*A*).^(^ ^17^ ^)^

As expected, in Ctsk‐Cre^+^:tdT mice, tdT was expressed in the fibroblasts of the periosteum and the suture=(Fig. 4*B‐a*). To identify the tdT^+^ cells as skeletal progenitors, we performed immunofluorescence staining with an anti‐Sp7/osterix antibody. The tdT^+^ cells were stained positively for Sp7 in the nuclei (empty white arrows), confirming them as skeletal progenitors (Fig. 4*B‐b*).

#### Identification of tdT ^+^ cells by IF

**Fig. 5:**
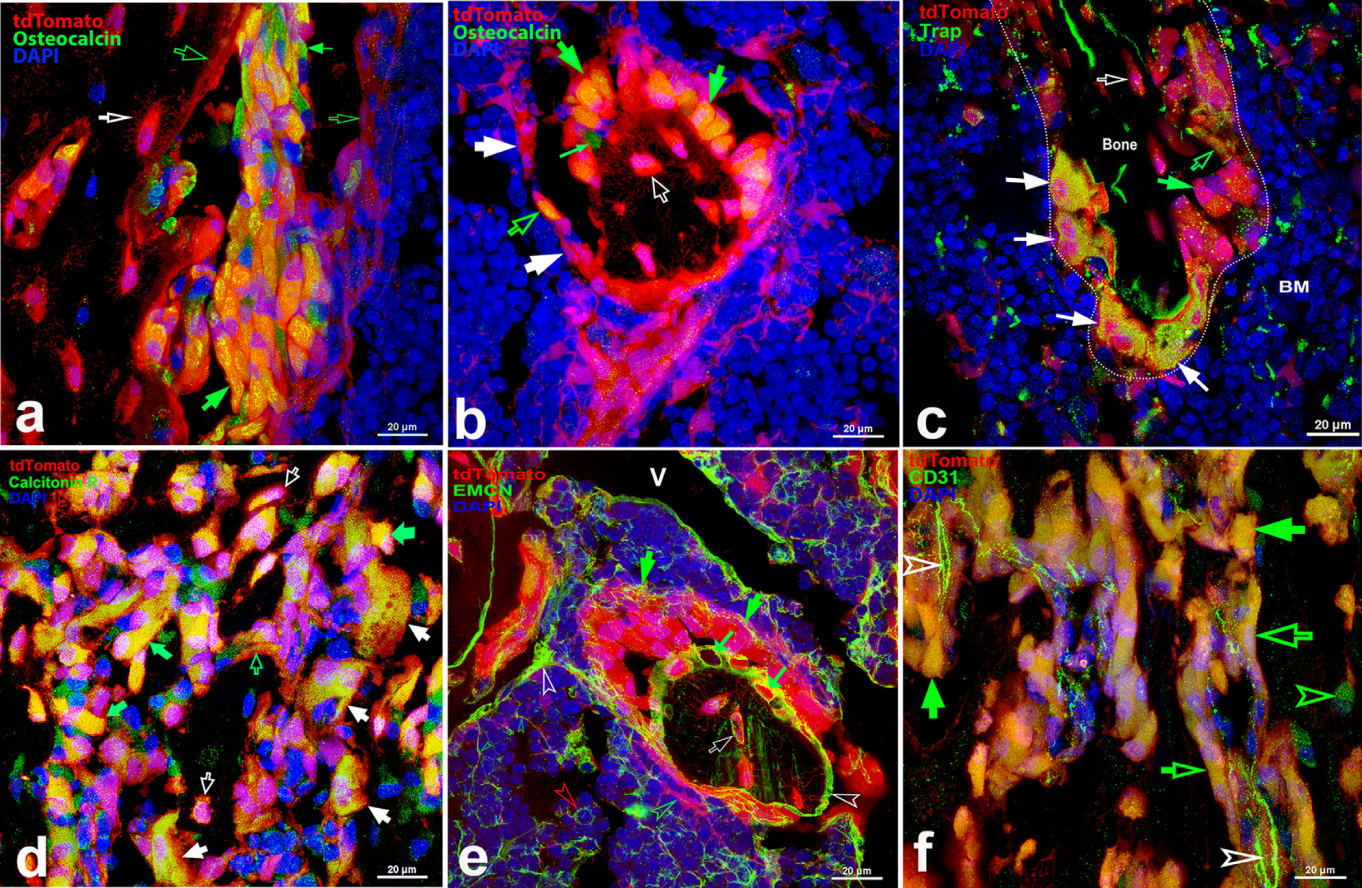
Identification of tdT^+^ cells by immunofluorescence staining. In femoral/tibial sections from Ctsk‐Cre^+^:tdT mice, a set of antibodies was applied to identify Ctsk^+^ cells, as described below. (*A*) Osteocalcin, showing that a cluster of tdT^+^ cells that stained positively for osteocalcin was osteoblasts in the epiphysis. (*B*) Osteocalcin, revealing that a group of cuboidal tdT^+^ cells that stained positively for osteocalcin was osteoblasts on the surface of trabecular bone within the bone marrow. (*C*) TRACP, showing multinucleated tdT^+^ cells stained positively for TRACP were osteoclasts. (*D*) Calcitonin R, illustrating that tdT^+^ cells stained positively for calcitonin R. (*E*) EMCN, demonstrating EMCN expression on bone surface, particularly areas covering surface of each individual cell. (*F*) CD31, indicating CD31 expression in type H vessels and osteoblasts located on bone surface of epiphysis. Arrows: green: cuboid‐shaped tdT^+^ cells are osteoblasts positive for osteocalcin; small green: cells positive for osteocalcin without tdT expression; white: multinucleated tdT^+^ cells in resorption bays that were positive for TRACP or calcitonin R were osteoclasts; empty white: osteocytes embedded within bone matrix; empty green: flat cells lining bone surface. V, vessel; BM, bone marrow. Scale bars, 20 μm. Magnification: ×1000.

To confirm the identities of the tdT^+^ cells described earlier, we performed immunofluorescence with antibodies against osteocalcin, Tracp, calcitonin receptor (R), EMCN (a mucin‐like sialoglycoprotein), or CD31 to recognize osteoblasts, osteoclasts, or endothelial cells (Fig. 5).

As shown, positive staining for osteocalcin was observed in the cuboid and nucleated tdT^+^ cells on the surface of epiphyseal bone and trabecula (green arrows), clearly demonstrating that these tdT^+^ cells were osteoblasts (Fig. 5*A,B*); in addition, flat tdT^+^ cells lining the bone surface or embedded within bone also showed positive staining (empty green and white arrows, respectively), confirming them as bone lining cells and osteocytes (Fig. 5*A.B*). However, a few cells showed positive staining for osteocalcin in the absence of tdT expression (small green arrows) (Fig. 5*A,B*).

To identify osteoclasts, we used two antibodies against TRACP and calcitonin R. Cells with more than two tdT^+^ nuclei were stained strongly for TRACP in the cytoplasm (white arrows), revealing these cells as osteoclasts (Fig. 5*C*), whereas cuboid tdT^+^ cells/osteoblasts and embedded osteocytes also showed slight staining for TRACP (green and empty white arrows, respectively) (Fig. 5*C*). In Fig. 5*D*, positive calcitonin R staining was observed in multinucleated tdT^+^ cells (white arrows), as well as nucleated tdT^+^ cells (osteoblasts and osteocytes) (green and empty white arrows, respectively). Calcitonin R staining was also found in tdT^+^ osteocytes in the sectioned cortical bone of the patella (Data S3 *A*).

To determine whether tdT was expressed in the vascular structure of bone, we applied two antibodies against EMCN (Fig. 5*E*) and CD31 (Fig. 5*F*). EMCN staining overlapped with small tdT^+^ vessels in the bone marrow (empty green arrowhead); EMCN also showed intense staining on the surface of trabeculae (white empty arrowheads). More interestingly, we found that EMCN staining was not detected in the cytoplasm but was strikingly adjacent to the membrane in a tdT^+^ cell‐like network, covering and separating the cells from each other (small green arrows) (Fig. 5*E*). Data S3 *B* shows EMCN staining in type H vessels in the epiphysis, but tdT was not expressed in the vessels; intriguingly, tdT^−^ chondrocytes in the growth plate were tightly enveloped by EMCN‐stained vessels (Data S3 *B*). In contrast, CD31 staining was observed not only in type H vessels in the epiphysis but also in cuboid cells (osteoblasts) on the bone surface (Fig. 5*F*).

#### Ctsk expression in cultures of osteoclasts and osteoblasts

To further investigate Ctsk expression in osteoblasts and osteoclasts, we cultured osteoblasts and osteoclasts using mBMSCs or bone marrow mononuclear cells derived from either Ctsk‐Cre^+^:nT‐nG or Ctsk‐Cre^+^:tdT mice.

**Fig. 6:**
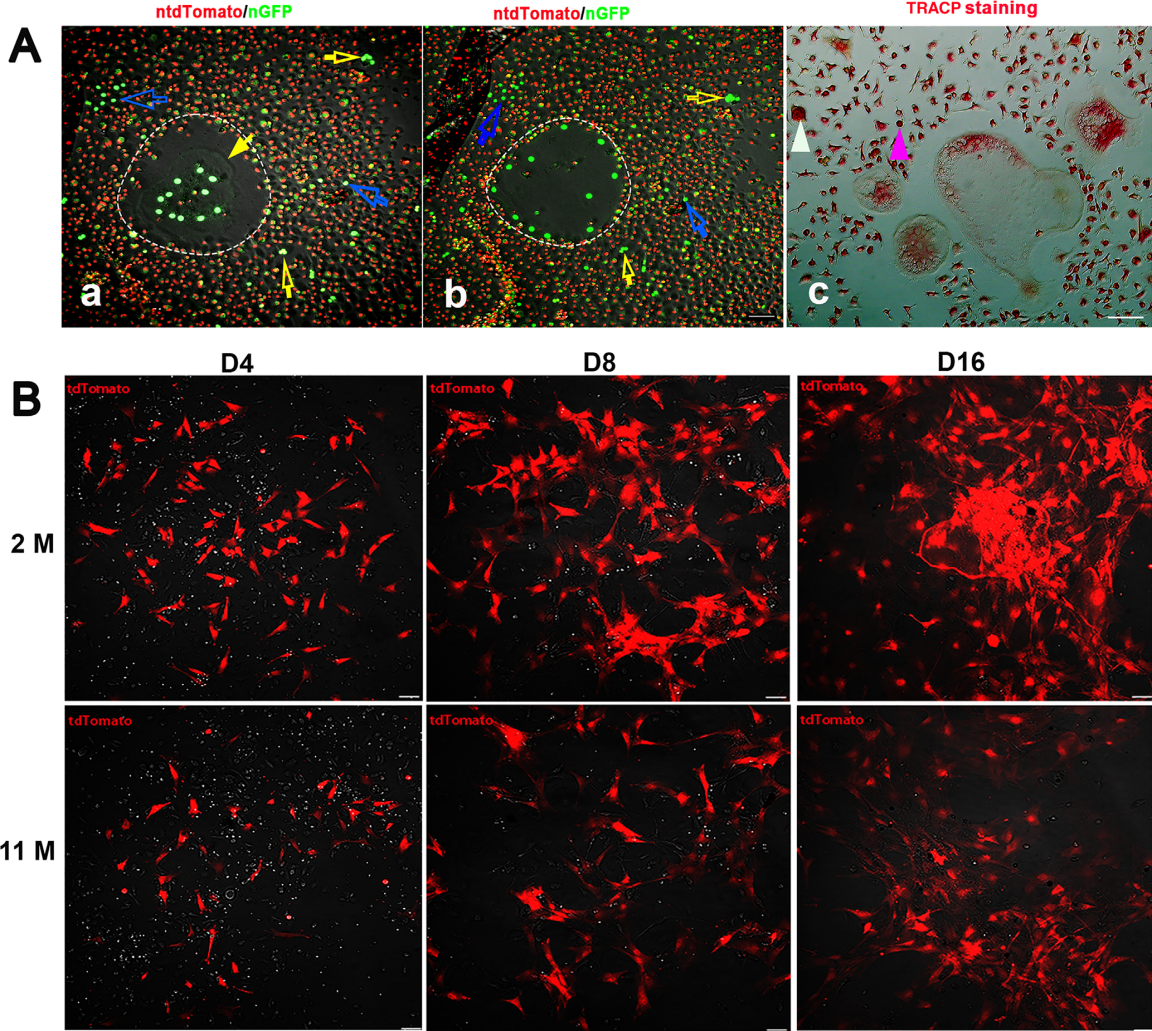
Ctsk^+^ cells in osteoclast or osteoblast cultures. Ctsk expression was examined in cultures of osteoclasts and osteoblasts. The mononuclear cells or mBMSCs from the BM were induced to differentiate into either osteoclasts (*A*) or osteoblasts (*B*). For osteoclast culture, bone marrow cells were harvested from Ctsk‐Cre^+^:nT‐nG mice aged 2 months and induced to differentiate into osteoclasts with M‐CSF and RANKL. In the early stage of osteoclast culture, the nGFP^+^ nuclei accumulated in the center of osteoclasts (*a*), and then these nuclei were found close to the cellular membrane after 2 days of further culture (*b*). In addition, nGFP^+^ was observed in small multinuclear osteoclasts (empty white arrows) and mononuclear cells (pink arrowheads). The yellow arrow indicates the nuclear membrane. At the end of osteoclast culture, TRACP staining was performed for 4% PFA‐fixed cells (*c*), showing Tracp^+^ staining of mononuclear cells (pink arrowhead) and small (white arrowhead) and large multinuclear osteoclasts. In osteoblast cultures, mBMSCs were derived from Ctsk‐Cre^+^:tdT mice aged 2 and 11 months (*B*). The tdT^+^ cells were detected on days 4, 8, and 16 during osteoblast differentiation culture. Scale bars, 50 μm. Magnification: ×200 for images in (*A*) and (*B*).

In osteoclast cultures from Ctsk‐Cre^+^:nT‐nG mice, bone marrow mononuclear cells were induced to differentiate into osteoclasts with both M‐CSF and RANKL. As expected, bright nGFP was observed in the nuclei of mature osteoclasts, which accumulated at the center of the nucleus in the early stage (yellow arrow indicates the nuclear membrane) and then moved near the cellular membrane with differentiation (Fig. 6*A‐a*). In addition, nGFP was also expressed in small multinucleated osteoclasts (empty yellow arrows) (Fig. 6*A‐b*). Surprisingly, nGFP was observed in every mononuclear macrophage (empty white arrows) (Fig. 6*A*). These cells were also positively stained for TRACP (Fig. 6*A‐c*).

During osteoblast differentiation, mBMSCs were harvested from Ctsk‐Cre^+^:tdT mice aged 2 and 11 months. As shown in Fig. 6*B*, interestingly, we observed tdT^+^ cells among colony forming unit‐fibroblasts (CFU‐fs) on day 4 and more tdT^+^ cells on days 8 and 16 during osteoblast culture, and more tdT^+^ cells were seen in the young than in the aging during the cultures (Fig. 6*B*).

### Ctsk‐Cre expression in nonskeletal tissues and organs

To assess Ctsk expression in tissues/organs other than bone, we examined adipose, lung, skeletal muscle, kidney, liver, pancreas, heart, testis, and brain tissues/organs (Fig 7).

**Fig. 7‐1:**
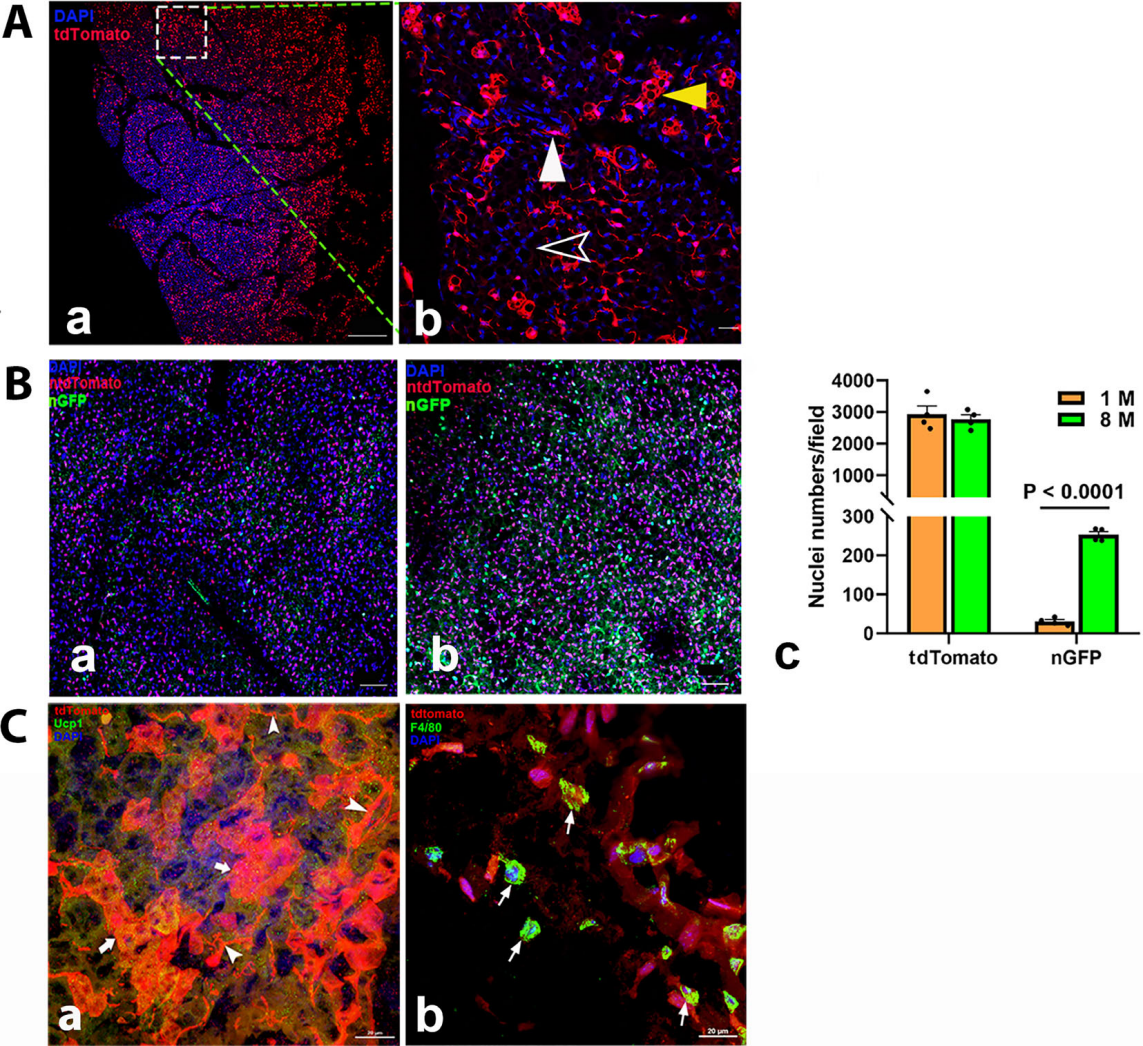
Ctsk^+^ cells in BAT. Ctsk^+^ cells were detected in either Ctsk‐Cre^+^:tdT (*A*) or Ctsk‐Cre^+^:nT‐nG (*B*) mice. The frozen sections were further stained with the Ucp1 antibody (*C*). In Ctsk‐Cre^+^:tdT mice aged 1 month (*A*), a representative area is amplified at higher magnification in the left panel. Arrowheads: white: arteriole; yellow: brown cells; empty white arrowhead: white adipose cells. In Ctsk‐Cre^+^:nT‐nG mice (*B*), the nT‐ and nG‐positive cells from the 1‐month (*a*) and 8‐month (*b*) groups and their comparison (*c*, *n* = 3). tdT^+^ cells were identified as brown cells by immunofluorescence staining with an anti‐Ucp1 antibody (*C‐a*), and macrophages were recognized by IF with an anti‐F4/80 antibody (*C‐b*). Arrows: wide white: tdT^+^ cells stained positively for Ucp1; white arrowheads: dendritic structure similar to peripheral nerve fibers; white: F4/80 positive staining cells without tdT expression. Scale bars, 50 μm for (*A*) and (*B*); 20 μm for (*C*). Magnifications: ×200 for (*A*) and (*B*); ×1000 for (*C*).

#### Adipose tissues

##### BAT

In Ctsk‐Cre^+^:tdT mice, tdT was seen in the small vessels and fibroblastic cells in the septa (Fig. 7‐1*A‐a*). At a high magnification, it was highly expressed in brown cells (yellow arrowhead) and weakly expressed in white adipose cells (empty white arrowhead); it was also expressed in an arteriole (white arrowhead) (Fig. 7‐1*A‐b*). Similarly, nGFP was found in brown cells and capillaries (Fig. 7‐1*B*). Although there was no difference in nT^+^ cell numbers between the young (aged 1‐month, Fig. 7‐1*B‐a*) and aging (aged 8‐months, Fig. 7‐1*B‐b*) groups, an increase in the number of bright nGFP^+^ cells was observed in the aging group (Fig. 7‐1*B‐c*).

**Fig. 7‐2:**
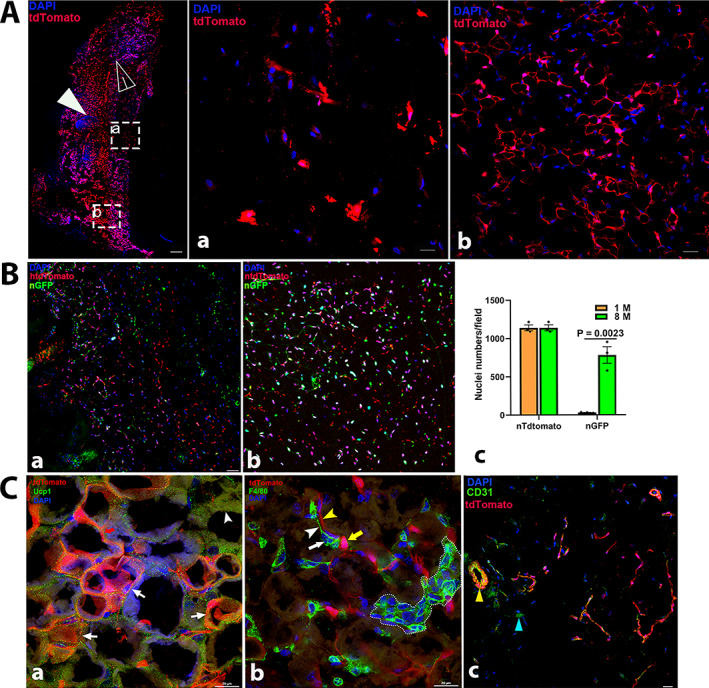
Imaging of Ctsk‐expressing cells in WAT.tdT^+^ cells in Ctsk‐Cre^+^:tdT mice (*A*) and Ctsk‐Cre^+^:nT‐nG mice (*B*). A piece of the WAT from Ctsk‐Cre^+^:tdT mice is shown at a magnification of ×40, with two areas in higher‐magnification images (×200): a: area containing more white adipocytes; b: area containing more beige cells. In Ctsk‐Cre^+^:nT‐nG mice, the Ctsk^+^ cells are recognized as nG+ cells derived from nT (*B*), with (*a*) 1‐month mice; (*b*) 8‐month mice, and (*c*) their comparison (*n* = 3), showing a remarkable increase in the number of nT+ cells during aging (*n* = 3). tdT^+^ beige cells were confirmed by IF with antibodies to identify beige cells, macrophages, and endothelial cells, respectively (*C*): (*a*) Ucp1, (*b*) F4/80, and (*c*) CD31. Arrows: white, staining of Ucp1 in tdT^+^ cells; white arrowhead, positive staining for only Ucp1 (*a*); white arrow: positive cells for F4/80; white arrowhead: F4/80‐stained fiber structure; yellow arrow: tdT^+^ cells, yellow arrowhead: dendrite‐shaped tdT^+^ cells (*b*); yellow arrowhead: arteriole; blue arrow: cells stained positive for CD31 (*c*). Scale bars, 500 μm for left panel of (*A*), 50 μm for *A*‐(*a, b*) and *B*‐(*a, b*); 20 μm for (*C*) magnifications: ×40 for left panel of (*A*); ×200 for *A*‐(*a, b*) and *B*‐(*a, b*); and ×1000 for (*C*).

##### WAT

tdT was seen in small blood vessels and dense areas with more beige cells in Ctsk‐Cre^+^:tdT mice (Fig. 7‐2*A*, the left panel), and two areas are shown at a high magnification: one showed weak tdT expression representative of adipocytes (Fig. 7‐2*A‐a*), and the other showed strong tdT expression representative of beige cells (Fig. 7‐2*A‐b*). In addition to similar expression in BAT, the numbers of nGFP^+^ cells were again found to be markedly increased with aging (Fig. 7‐2*B*).

**Fig. 7‐3:**
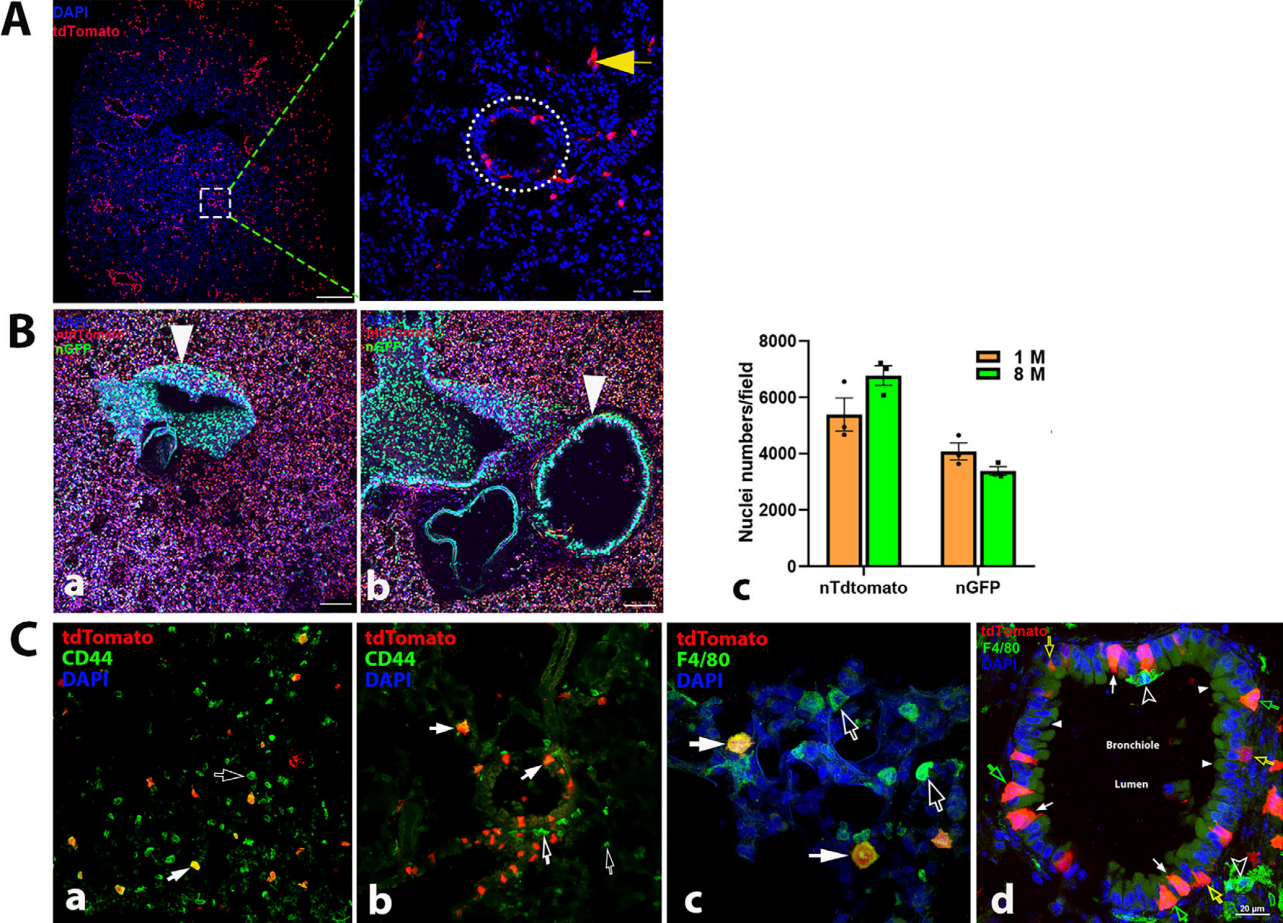
Representative images of Ctsk expression in lung. tdT+ cells were examined in lung sections of Ctsk‐Cre+:tdT mice (*A*). tdT was highly expressed in certain cells in alveoli and bronchioles (*a*), and a representative area is shown at a higher magnification (*b*). Arrows: yellow: type II alveolar cells or macrophages in alveoli. A dotted circle indicates a bronchiole. In Ctsk‐Cre+:nT‐nG mice (*B*), quantitative analysis of nT and nG expression was performed in young (*a*) and aged (*b*) mice (*c*, *n* = 3). White arrowhead: bronchioles. Antibodies against either CD44 or F4/80 were used to identify macrophages or type II pneumocytes by IF (*C*), showing CD44 in alveoli (*a*) and bronchioles (*b*); F4/80 in alveoli (*c*); F4/80 in cells adjacent to bronchioles (*d*). Arrows: in (*C‐a‐c*) empty white: cells that only stained positively for CD44 but not tdT expression were macrophages; white: CD44‐ or F4/80‐stained cells with tdT expression were type II pneumocytes or macrophages; in (*d*) white: Clara secretory cells/Goblet cells, mucus‐producing cells; white arrowhead: ciliated cells; empty yellow: basal cells; empty green: neuroendocrine cells/putative early progenitors; empty white arrowheads: macrophages. AD, alveolar duct; AS, alveolar sac; *A*, alveoli. Scale bars, 500 μm for (*C‐a*), 50 μm for (*A*, *B*); and 20 μm for (*C‐c*,*d*). Magnifications: 200 for (*A*) and (*B*); 200 for (*A*, *B*, *C‐a*); 400 for (*C‐b*); 1000 for (*C‐c*,*d*).

In addition, we performed immunofluorescence with antibodies against Ucp1 or F4/80 to determine whether the tdT^+^ cells were brown/beige cells and to distinguish brown cells from macrophages. Both tdT^+^ cells in BAT and WAT showed positive staining for Ucp1, as indicated by white arrows, demonstrating that these cells were brown and beige cells (Figs. 7‐1*C‐a* and 7‐2*C‐a*). Unexpectedly, tdT was not expressed in F4/80‐stained macrophages (white arrows) (Figs. 7‐1*C‐b* and 7‐2*C‐b*). We further stained the WAT sections with an anti‐CD31 antibody and found that it was positively stained in endothelial cells of the arteriole, whereas tdT was expressed in the smooth muscles of the arteriole (yellow arrowhead) (Fig. 7‐2*C‐c*).

As we observed tdT^+^ nerve fiber‐shaped structures in both BAT and WAT, we then stained the BAT sections with an antibody against tyrosine hydroxylase (TH) to define them. We found that TH‐positive nerve fibers/axons were surrounded by the tdT^+^ sheath in large nerve fibers, indicated by blue and white arrows, respectively (Data S4 *A*‐a,b, and S4*B*‐a–c). However, small never fibers or terminal fibers were only partially colocalized with tdT^+^ fiber structures (Data S4 *C*‐a–c), and further investigation is needed. Surprisingly, we also observed that TH was highly expressed in tdT^+^ browning cells at low and high magnifications. Both tdT^+^ and TH‐stained cells are indicated by empty arrowheads, whereas browning cells stained only for TH without tdT expression are indicated by white arrowheads (Data S5 *A,B*).

#### Lung

We observed both tdT^+^ and nG^+^ cells in the alveoli and bronchioles (Fig. 7‐3*A,B*). However, no difference in nG was found between the aging mice and the young mice (Fig. 7‐3*B‐c*). To clarify whether tdT^+^ cells were macrophage/type II pneumocytes, we stained tdT^+^ sections with CD44 and F4/80 antibodies and observed CD44‐ and F4/80‐positive staining in tdT^+^ cells located in alveoli and nearby bronchioles (Fig. 7‐3*C‐a–c*). Interestingly, tdT was co‐expressed in CD44‐ or F4/80‐positive cells but not in all of the CD44‐ and F4/80‐positive cells (Fig. 7‐3*C‐a–c*). In the bronchiole, tdT^+^ cells were recognized as Clara secretory cells/Goblet cells, mucus‐producing cells, basal cells, and neuroendocrine cells/putative early progenitors based on their morphologies and distributions in the bronchiole at high magnification (Fig. 7‐3*C‐c*).^(^ ^32^ ^)^

**Fig. 7‐4:**
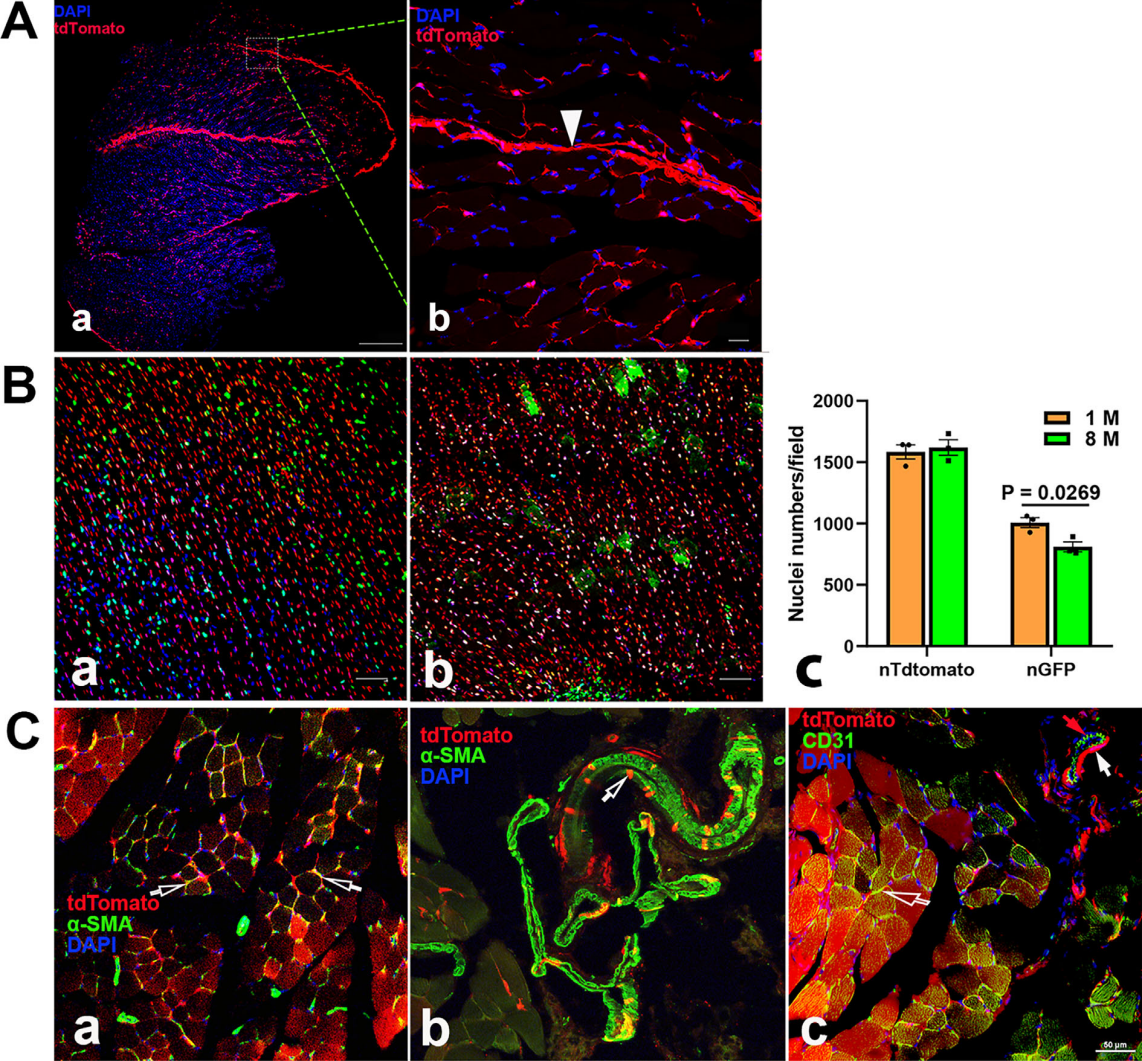
Imaging of Ctsk expression in skeletal muscle sections. tdT expression was detected in Ctsk‐Cre^+^:tdT mice (*A*). An image of a lobe of muscle fibers at low magnification is presented (*a*). A representative area is shown at a high magnification (*b*), showing strong tdT expression in the connective tissue, whereas lower tdT expression was observed in the sarcoplasm of muscle fibers. White arrowhead: perimysium. In Ctsk‐Cre^+^:nT‐nG mice (*B*), Ctsk expression is recognized in nG^+^ cells switched from nT^+^ cells in mice aged either 1 month (*a*) or 8 months (*b*). The expression of nT‐ and nG‐positive cells was quantitatively analyzed (*c*) (*n* = 3), showing a significant reduction in nG expression in the 8‐month‐old mice. Immunofluorescence was performed with antibodies against α‐SMA and CD31 to identify satellite cells and smooth muscle of the arteriole (*C*). (*a*) Cells positive for both tdT and α‐SMA in skeletal muscle; (*b*) cells positive for both α‐SMA and tdT in artery; and (*c*) cells positive for CD31 in muscle adjacent to artery. Arrows: empty white: satellite cells in (*a*) and (*c*); empty white: cells positive for both tdT and α‐SMA of artery in (*b*); white: smooth muscle, red: internal elastic lamina/tunica intima in (*c*). Scale bars, 500 μm for left panel of (*A*), 50 μm for (*B, C*); and 20 μm for right panel of (*A*). Magnifications: ×40 for left panel of (*A*); ×200 for (*B*); and ×400 for right panel of (*A*) and (*C*).

#### Skeletal muscle

In transverse sections of skeletal muscles from young mice (1 month), we observed strong tdT staining in the endomysium and perimysium (Fig. 7‐4*A‐a*); tdT was also found in cells around the muscle fibers, which were morphologically similar to satellite cells, with weak expression in the sarcoplasm of muscle fibers at a high magnification (Fig. 7‐4*A‐b*). Compared to that in young mice, a remarkable increase in tdT expression was observed with aging (Figs. 1 and 2), in contrast to the decrease in nG observed in Ctsk‐Cre^+^:nT‐nG mice (Fig. 7‐4*B*).

**Fig. 7‐5:**
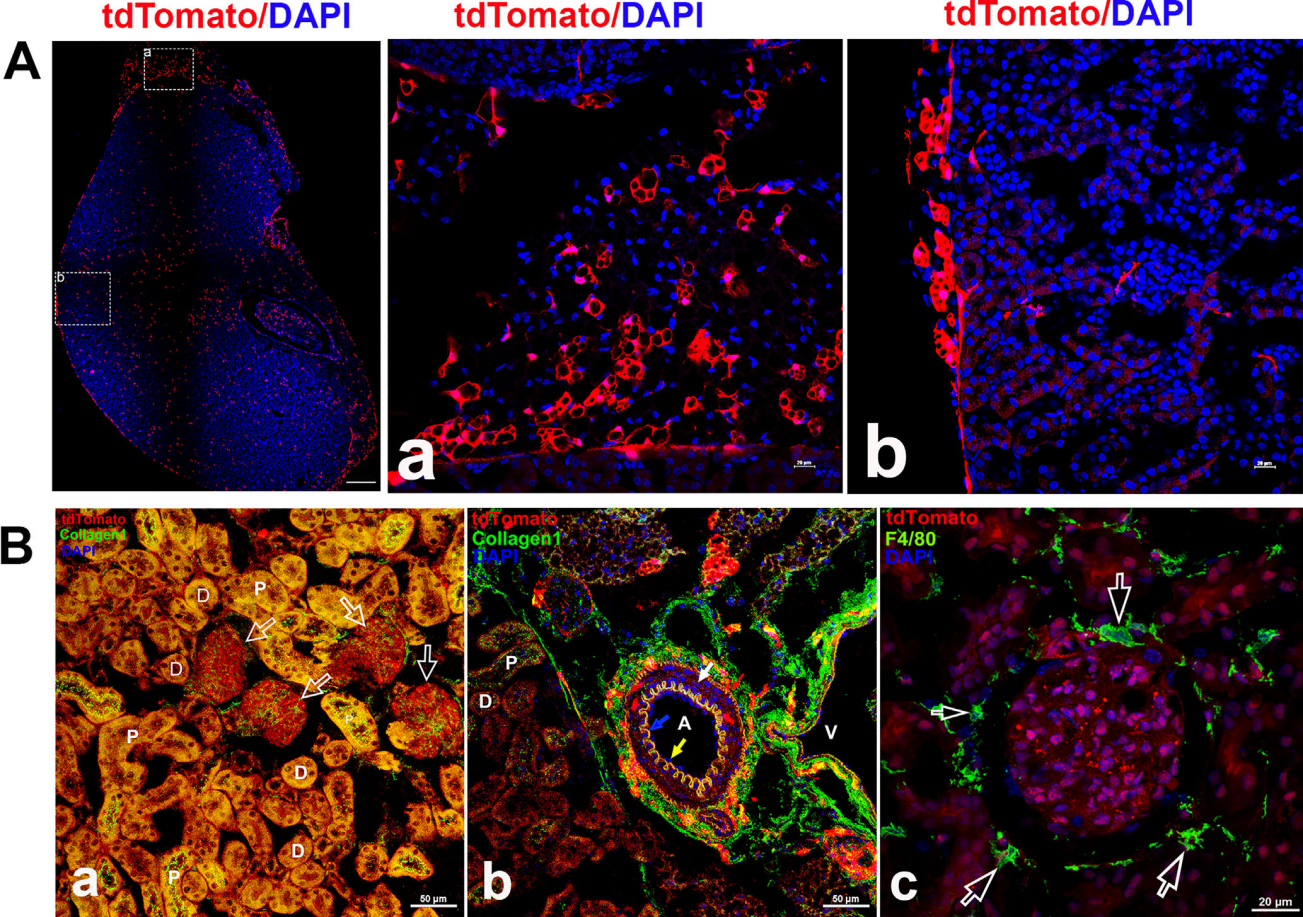
Representative images of Ctsk expression in kidney. Ctsk expression in Ctsk‐Cre+:tdT mice at low magnification (40) (*A*). Whole kidney image is shown in left panel with two representative areas: Ctsk expression in adrenal gland (*a*) and renal cortex with fibrous capsule (*b*). Immunofluorescence was carried out with antibodies against type I collagen and F4/80 in renal parenchyma. The results are shown in (*B*): a: type I collagen staining; b: type I collagen staining in arteriole; and c: F4/80 staining. P, proximal convoluted tubule; D, distal convoluted tubules; A, arteriole; V, vein. Arrows in (*B‐a*): empty white arrows: renal corpuscles; in (*b*): yellow: endothelial cells; red: outer elastic layer (Tunica externa); white: smooth muscle layer (Tunica media); blue: inner elastic layer (Tunica intima); in (*c*): white empty: positive staining for F4/80. Scale bars, 500 μm for whole kidney image and 20 μm for (*A‐a*, *b*); 50 μm for images for (*B*) and (*C‐a*); 20 μm for (*C‐b*–*d*). Magnifications: 40 for whole kidney and 400 for representative images in (*A‐a*, *b*), and (*B‐c*). 1000 for images in (*a*), (*b*) and (*d*) in (*C*).

To clarify the identities of tdT^+^ cells around the muscle fibers and in blood vessels, we further imaged the sections after staining with antibodies against α‐SMA or CD31. The results showed that the tdT‐positive cells around the fibers were satellite cells (empty white arrows), as they showed positive staining for α‐SMA, and tdT expression was found in the α‐SMA‐stained vascular smooth muscle (empty white arrow) (Fig. 7‐4*C‐a,b*). In addition, tdT^+^ satellite cells also showed positive staining for CD31 (empty white arrow) (Fig. 7‐4*C‐c*). In the arterioles, CD31 staining was found only in the elastic lamina (red arrow) but not in tdT^+^ smooth muscle (white arrow) (Fig. 7‐4*C‐d*).

#### Kidney

In Ctsk‐Cre^+^:tdT mice, tdT expression was observed in the adrenal gland and the fibrous capsule covering the kidney (Fig. 7‐5*A*), as well as in renal tubules and glomeruli (Fig. 7‐5*A,B*). Immunostaining with collagen 1 antibody showed strong collagen 1 staining in both tdT^+^ renal tubes but less staining in the glomerulus (Fig. 7‐5*B‐a*), whereas in an arteriole, tdT was found in the outer elastic layer and smooth muscles (Fig. 7‐5*B‐b*). However, F4/80 staining demonstrated that F4/80 was not colocalized with tdT in the tube area or glomerulus (Fig. 7‐5*B‐c*), indicating that tdT is not expressed in macrophages.

**Fig. 7‐6:**
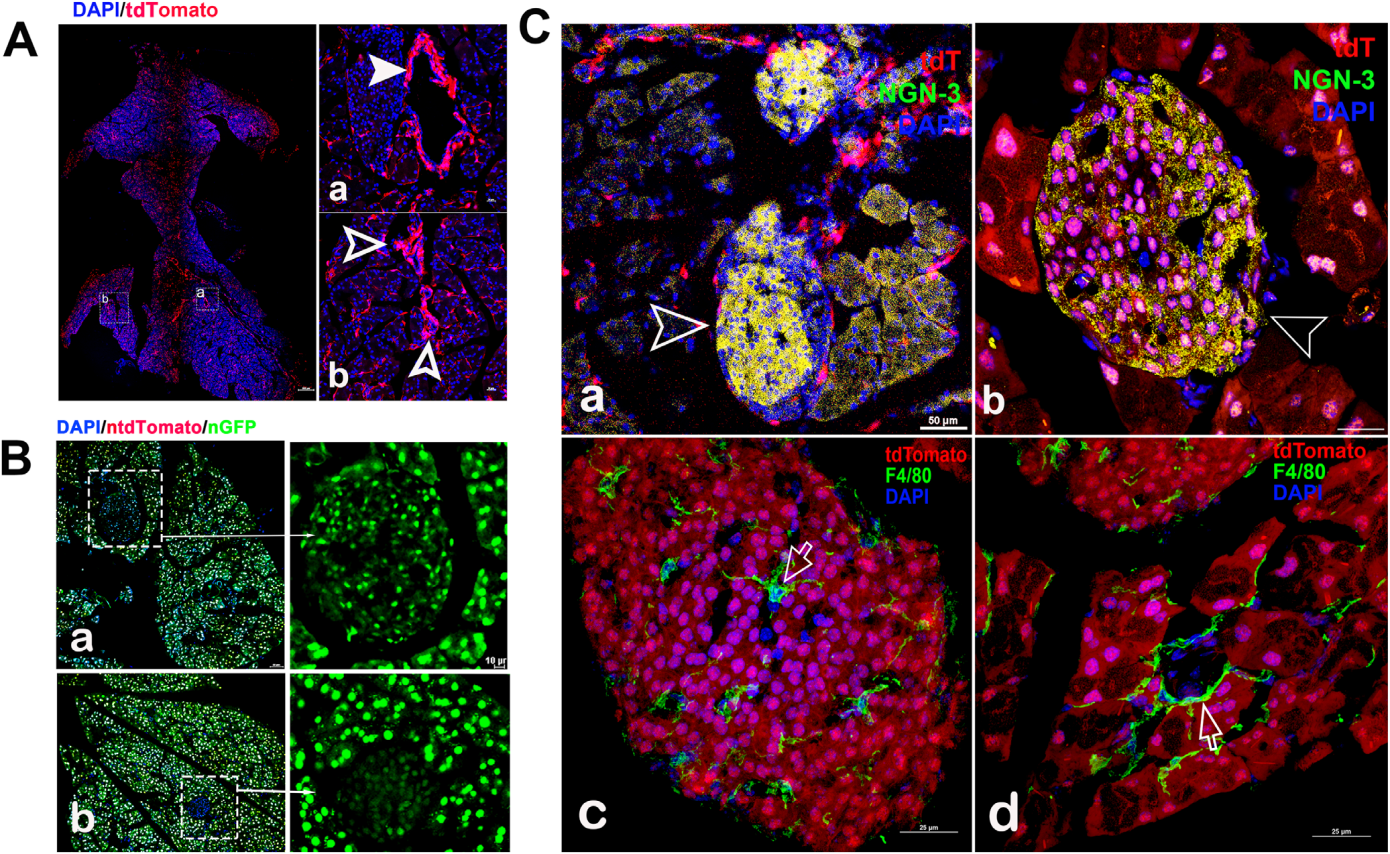
Detection of Ctsk expression in pancreas. In Ctsk‐Cre^+^:tdT mice (*A*), tdT was highly expressed in blood vessels (white arrowheads) and islets (white empty arrowheads), but it was weakly expressed in acinar cells. The left panel of (*A*) is an image of a pancreas section at a low magnification (×40). Representative images: (*a*) blood vessel (white arrowhead); (*b*) two islets (empty white arrowheads). In Ctsk‐Cre^+^:nT‐nG mice, Ctsk expression is indicated by nG (*B*). Its expression was examined in the mice aged 1 month (*a*) and 8 months (*b*) and their comparison (*c*), in which strong expression was detected in the islets of Langerhans in the young mice, but its expression was reduced in the aging mice. Immunofluorescence with antibodies against NGN3 or F4/80 was carried out to verify tdT^+^ cells or macrophages (*C*). tdT^+^ cells were identified as endocrine cells in the islets in the pancreatic islets of Langerhans, as they were positively stained for NGN3 (*a*: lower magnification and *b*: higher magnification; empty white arrowheads: islets). The F4/80‐stained cells were macrophages (*c*, an islet; *d*, an acinar area near the islet; empty white arrows‐macrophages). Scale bars as indicated in the images, 500 μm for the left panel of (*A*) and 20 μm for (*A‐a, b*); 50 μm for (*B‐b*); and (*C‐a*); 20 for (*C‐b–d*); and 10 for right panels of (*B*). Magnifications: ×40 for left panel in (*A*), ×200 for (*A‐a, b*), and (*B‐a, b*); ×400 for representative images of islets; ×1000 for (*C‐b–d*).

#### Pancreas

In the pancreas, high tdT expression was observed in the islets and blood vessels (white arrowhead), but low expression was observed in acinar cells (Fig. 7‐6*A*). nGFP was expressed in both islet and acinar cells, and a decrease in expression was observed in the islets of Langerhans during aging (Fig. 7‐7*B*, a: young, b: aging).

**Fig. 7‐7:**
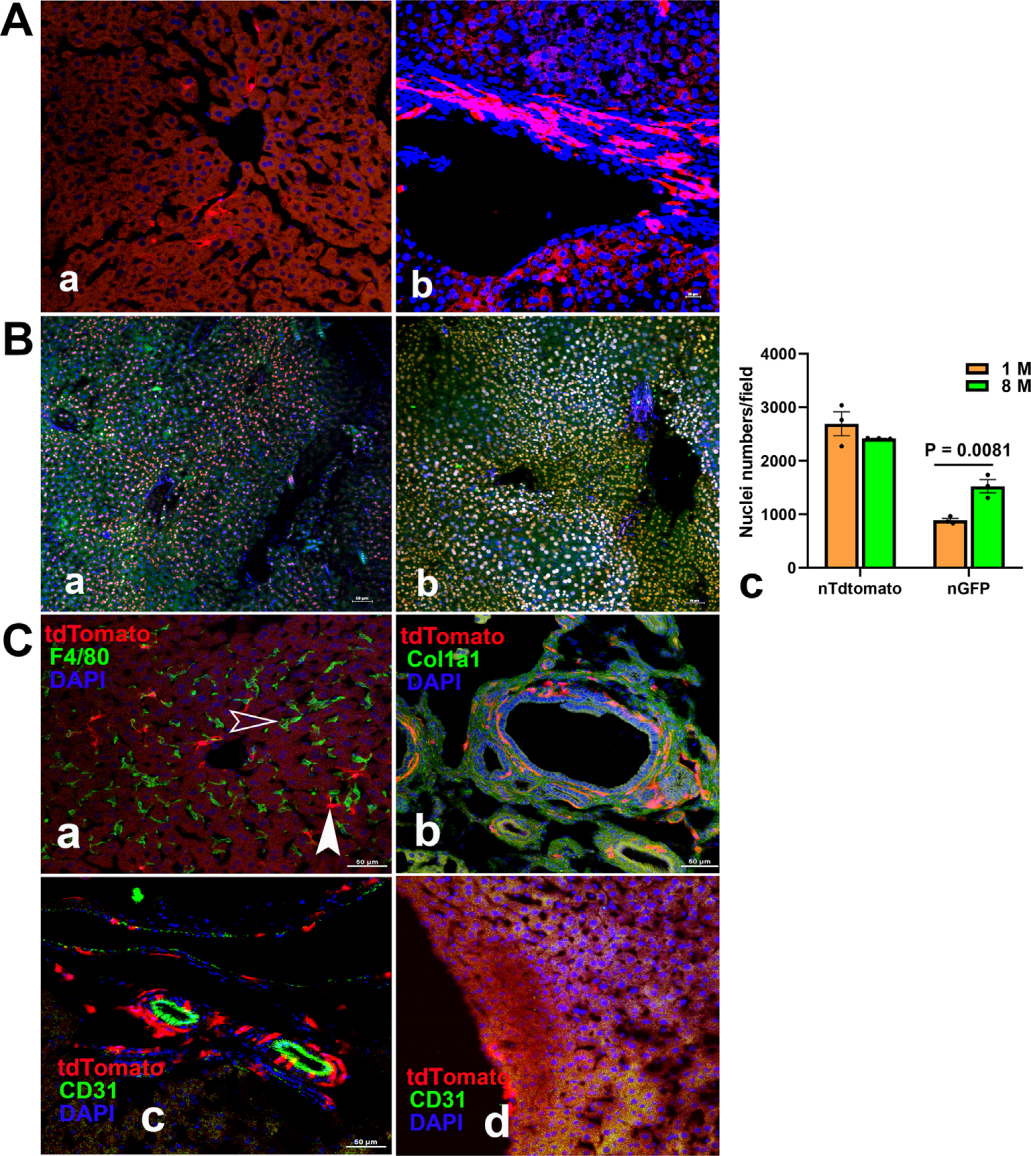
Fluorescence images of liver. tdT expression in Ctsk‐Cre^+^:tdT mice (*A*). Although tdT was expressed at low levels in hepatocytes (*a*), it was highly expressed in interlobular connective tissue (*b*). Ctsk expression in Ctsk‐Cre^+^:nT‐nG mice (*B*), aged at 1 month (a) and 8 months (*b*), with their comparison (*c*) (*n* = 3). nG was coexpressed with nT in hepatocytes and was significantly increased in aging mice. Immunofluorescence was performed to identify macrophages and endothelial cells with antibodies against F4/80 (*a*), Col1a1 (*b*‐artery), or CD31 (*c*: arterioles and *d*: hepatocytes located at edge of liver lobe), respectively. Arrowheads in (*C‐a*): empty white indicates cells stained positively for F4/80 but no tdT expression; white indicates cells expressing tdT but no F4/80 staining. Scale bars, 20 μm for (*A*); 50 μm for (*B*) and (*C*). Magnifications, ×400 for (*A*) and (*C*); ×200 for (*B*).

**Fig. 7‐8:**
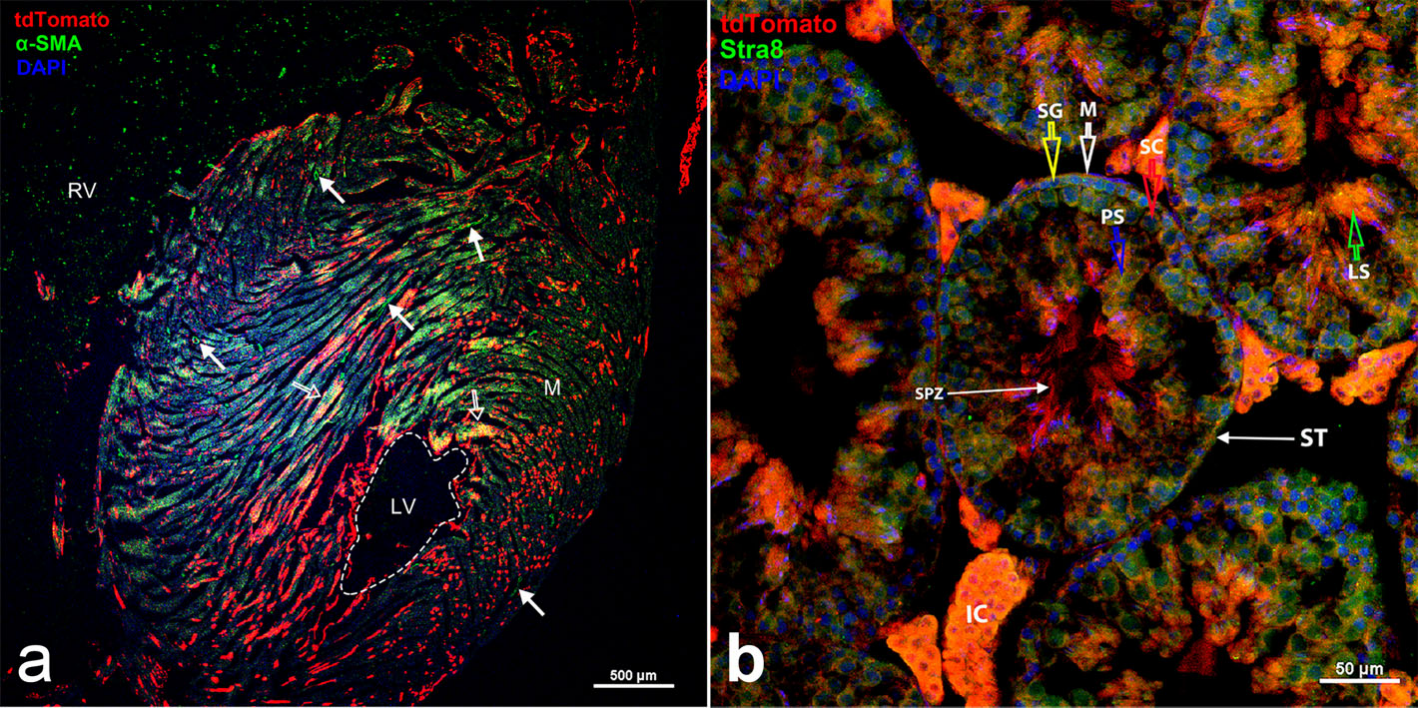
Ctsk expression detected in heart and testis. Ctsk expression was imaged in myocardium of Ctsk‐Cre^+^:tdT mice (*a*). Its expression was detected in cardiac muscle fibers. It was also co‐expressed with α‐SMA by immunofluorescence with an antibody against α‐SMA (indicated by empty white arrows). In addition, tdT expression was found in the coronary arteries, with α‐SMA expression (indicated by white arrows). RV, right ventricle; LV, left ventricle; M, myocardium. In addition, Ctsk expression was detected in testis sections (seminiferous tubules) from Ctsk‐Cre^+^:tdT mice (*b*). Its expression was localized in the interstitial tissue (IC of Leydig cells) and the seminiferous tubules (ST). It was also found in developing spermatozoa (SPZ). Arrows: yellow, spermatogonia (SG); white, myeloid cells (M); red, Sertoli cells (SC); and green, late spermatids (LS). Scale bar: 500 μm for and 50 μm for (*b*). Magnification: (*a*) ×40 and (*b*) ×400.

tdT^+^ cells in the islets were further verified as endocrine cells by NGN3 immunostaining at low and high magnifications (empty white arrowheads) (Fig. 7‐6*C‐a,b*). In addition, we determined that tdT^+^ cells were not macrophages by F4/80 immunostaining; F4/80‐stained macrophages are indicated by empty white arrows (Fig. 7‐6*C‐a,d*).

#### Liver

Although tdT was observed to be weakly expressed in hepatocytes (Fig. 7‐7*A‐a*), its expression was strong in the portal venule, hepatic artery (Fig. 7‐7*A‐b*), and cells morphologically similar to Kupffer cells/macrophages (Fig. 7‐7*A‐a*). In addition, the number of nGFP^+^ cells dramatically increased in the aging mice (Fig. 7‐7*B‐b*) compared to the young mice (Fig. 7‐7*B‐a,c*).

To determine whether tdT was expressed in macrophages, F4/80 immunostaining was carried out. On the one hand, the staining successfully identified macrophages (empty arrowhead), but no tdT expression was seen in these cells. On the other hand, tdT^+^ dendritic cells showed negative staining for F4/80 (white arrowhead). Therefore, tdT^+^ dendritic cells are not macrophages or Kupffer cells (Fig. 7‐7*C‐a*).

In the arterioles, Col1a1 staining was observed in the connective tissue layer and in smooth muscle, which also had tdT expression (Fig. 7‐7*C‐b*). In addition, CD31 staining clearly showed positive staining in the tdT^−^ elastic laminae (tunica intima) but not in tdT^+^ smooth muscle (Fig. 7‐7*C‐c*). Interestingly, at the edge of the liver lobe, we found strong CD31 staining in tdT^+^ hepatocytes (Fig. 7‐7*C‐d*).

#### Heart and testis

Fluorescence images showed tdT expression in the cardiac muscle fibers, as confirmed by α‐SMA immunostaining (empty white arrows), while the coronary arteries are indicated by white arrows (Fig. 7‐8*A*). In addition, tdT expression was observed in the seminiferous tubules (Fig. 7‐8*B*), located in the interstitial tissue (IC of Leydig cells), seminiferous tubules (ST), and developing spermatozoa (SPZ). Stra8 immunostaining confirmed the identities of the tdT^+^ cells as testis cells (Fig. 7‐8*B*).

**Fig. 7‐9 (A and B):**
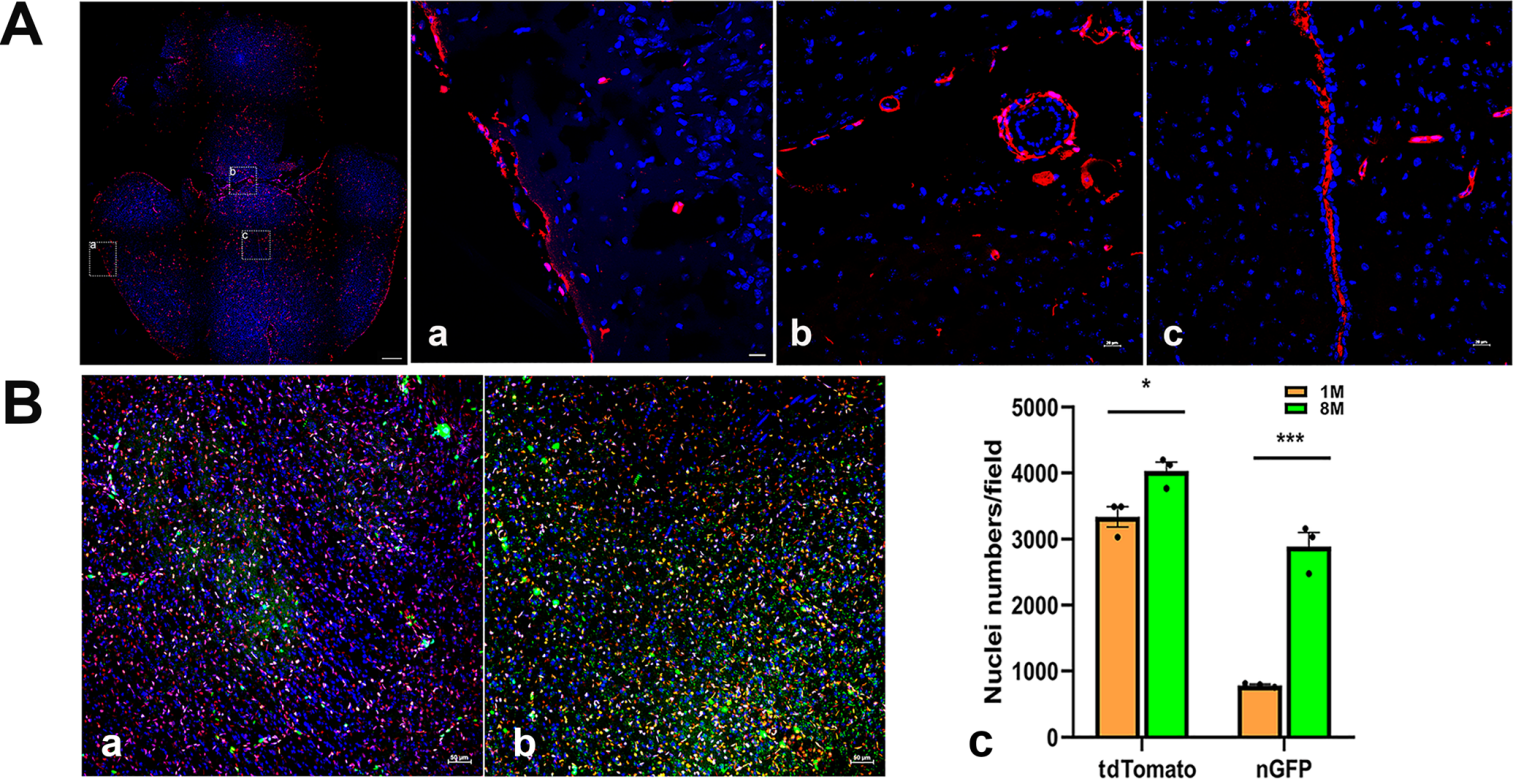
Ctsk expression in horizontal sections of brain. Ctsk^+^ cells imaged in whole‐brain horizontal section of Ctsk‐Cre^+^:tdT mice (left panel, low magnification) with three representative areas at higher magnification (*A*): (*a*) meninge; (*b*) blood vessel; and (*c*) falx cerebri in longitudinal fissure. Ctsk expression in Ctsk‐Cre^+^:nT‐nG mice (*B*), aged 1 month (*a*) and 8 months (*b*) and their comparison (*c*) (*n* = 3). Scale bars: 500 μm for whole‐brain image in left panel of (*A*); 50 μm for images in (*A‐a–c, B*). Magnifications: ×40 for left panel of (*A*); ×200 for (*A‐a–c, B*).

#### Brain

In this work, anatomic locations in neuroimages of horizontal brain sections were determined/referenced with high‐resolution 3D Allen Brain Reference Atlases (https://mouse.brain-map.org/ ).^(^ ^33^ ^)^ Based on this information, a schematic map for the locations of neuroimages in this study is shown in the Supplemental Material.

In Cstk‐Cre^+^:tdT mice, we observed tdT^+^ cells in the meninges (Fig. 7‐9*A‐a*), blood vessels (Fig. 7‐9*A‐b*), and falx cerebri of the longitudinal fissure (Fig. 7‐9*A‐c*). In addition, small tdT^+^ cells were observed throughout the whole brain at low magnification (Fig. 7‐9*A*, right panel). In Cstk‐Cre^+^:nT‐nG mice, the numbers of both nT^+^ and nG^+^ cells were significantly increased in aging mice (Fig. 7‐9*B‐b*) compared to young mice (Fig. 7‐9*B‐a,c*).

**Fig. 7‐9 (C):**
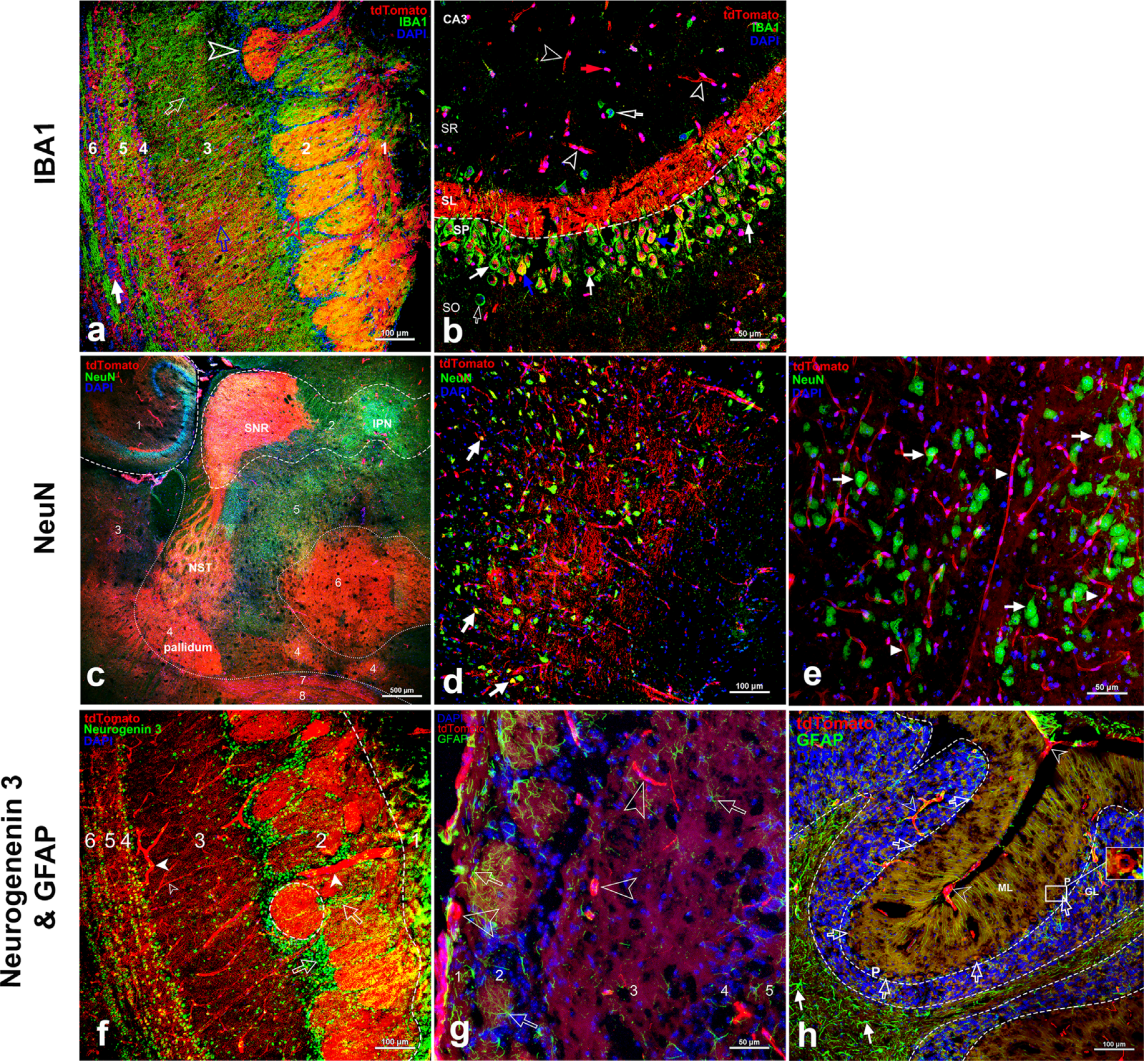
(*C*) Identification of tdT^+^ cells by immunofluorescence with antibodies against IBA1 (a marker of microglia) (*a, b*), NeuN (neuronal nuclei, a marker of CNS neuronal cells) (*c, d, e*), Neurogenin 3 (neurogenic astrocytes and oligodendrocytes) (*f*), and GFAP (a marker for astrocytic cells) (*g, h*) in the following areas: (*a*) in the olfactory bulb, immunofluorescence with an IBA1 antibody. Arrows: white: tdT^+^ cells in sixth layer (anterior commissure); empty arrowhead: OB with strong tdT expression and colocalized IBA1 staining. Layers: 1: glomerular layer; 2: external plexiform layer; 3: mitral cell layer; 4: internal plexiform layer; 5: granule cell layer; 6: anterior commissure. Scale bar: 100 μm. Magnification: ×200. (*b*) In hippocampus (Cornu ammonis 3, CA3), immunostaining with IBA1 antibody. Arrows: white arrows: pyramidal cells positive for both tdT (nuclei) and IBA1 (cell body); empty white arrows: positive for IBA1; and empty arrowheads: blood vessels or neural dendrites. SR, strata radiatum; SL, strata lucidum; SO, stratum oriens; SP, stratum pyramidale layers. Scale bar: 50 μm; Magnification: ×400. (*c*) In several areas, staining with NeuN antibody at lower magnification. Areas: 1: hippocampus; 2: midbrain; 3: striatum; 4: pallidum; 5: hypothalamus; 6: thalamus; 7: anterior commissure tempered limb; 8: anterior commissure olfactory limb. IPN, interpeduncular nucleus; SNR, substantia nigra reticular part; NST, nigrostriatal tract. Scale bar: 500 μm. Magnification: ×40. (*d*) In medulla, staining with NeuN antibody. Arrows: white: motor neurons; empty arrowhead: blood vessels. Scale bar: 100 μm. Magnification: ×200. (*e*) In pons, staining with NeuN antibody. Arrows: white: neural cells positive for NeuN; empty arrowheads: blood vessels. Scale bar: 50 μm. Magnification: ×400. (*f*) Staining with Neurogenin 3 antibody was performed in olfactory bulb. Arrows: empty white: neural cells stained positive for Neurogenin 3 (periglomerular cells); white empty arrowheads: blood vessels. Layers: 1: olfactory nerve layer; 2: glomerular layer; 3: external plexiform layer; 4: mitral cell layer; 5: internal plexiform layer; 6: granule cell layer. Scale bar: 100 μm. Magnification: ×200. (*g*) In olfactory bulb, staining with GFAP antibody. Arrows: empty white arrows: cells positive for GFAP (astrocytes); empty white arrowheads: blood vessels. Layers: 1: olfactory nerve layer; 2: glomerular layer; 3: external plexiform layer; 4: mitral cell layer; 5: internal plexiform layer. Scale bar: 50 μm. Magnification: ×400. (*h*) In cerebellum, staining with GFAP antibody. Arrows: white: cells positive for GFAP (astrocytes); empty white arrows: Purkinje neurons (P) positive for both tdT and GFAP; empty white arrowheads: vascellum. White square indicating a P cell with an amplified image shown on left. Layers: ML, molecular layer; GL, granular layer. Scale bar: 100 μm. Magnification: ×200.

To clarify tdT^+^ cell identities, we performed immunofluorescence with antibodies against IBA1, NeuN, or GFAP that recognize microglia, neurons, and astrocytes, respectively, in horizontal brain sections. The representative images are as described in what follows.

1. IBA1 for microglia
  - In the olfactory bulb (Fig. 7‐9*C‐a*), high tdT expression was seen in most olfactory bulbs, axon/fiber structures in the glomerular layer and mitral cell layer, neuron cell bodies, and axons in layers of the internal plexiform, granule cells, and the anterior commissure. In addition, microglial cells were identified by IBA1 immunostaining, but they did not express tdT.
  - In the hippocampus (cornu ammonis 3, CA3) (Fig. 7‐9*C‐b*), bright tdT expression was observed in the stratum lucidum (SL). Interestingly, tdT was found in the nuclei of pyramidal cells located in the stratum pyramidale (SP) that showed positive staining for IBA1 in the cell body, as indicated by white arrows. A few tdT^+^ cells were also seen in the layers of the stratum radiatum (SR) and stratum oriens (SO) (indicated by empty white arrowheads) (Fig. 7‐9*C‐b*).
2. NeuN for neural cells
  - At low magnification, tdT expression was observed in several areas, including the hippocampus, midbrain, striatum, pallidum, hypothalamus, thalamus, anterior commissure tempered limb, and anterior commissure olfactory limb (Fig. 7‐9*C‐c*). Higher tdT expression was observed in the substantia nigra reticular (SNR) part, nigrostriatal tract (NST), pallidum, and thalamus, whereas NeuN‐positive staining was noticed in the first three areas, as well as in the hippocampus, midbrain, and hypothalamus; however, tdT expression was undetectable in the interpeduncular nucleus (IPN).
  - In the medulla, NeuN staining distinguished neural cells from tdT^+^ fiber structures (blood vessels or neural axons). However, a few tdT^+^ cells were stained positively for NeuN, as indicated by white arrows in this region (Fig. 7‐9*C‐d*).
  - In the pons, immunofluorescence identified neural cells, in which tdT was undetectable; however, tdT expression was observed in the axon‐shaped structures (Fig. 7‐9*C‐e*).

In addition, the results of NeuN staining in other brain areas, including the olfactory bulb, cerebral cortex, thalamus, hypothalamus, and medulla, are shown in Data S6.

3 Neuogenin 3

As neuogenin 3, a basic helix–loop–helix (bHLH) transcription factor, is involved in neurogenesis,^(^ ^34^ ^)^ we used its antibody to recognize neural cells in the olfactory bulbs. Cells showed positive staining in all layers, especially in cells around the bulbs. However, tdT expression was not detected in these cells. Nevertheless, tdT was noted in tube‐/axon‐shaped structures (Fig. 7‐9*C‐f*).

4 GFAP for astrocytes

We also identified astrocytes by GFAP staining in the olfactory bulb and cerebellum (Fig. 7‐9*C‐g,h*). In the first area, no tdT expression was detected in the astrocytes (empty white arrows, whereas empty white arrowheads point to tdT^+^ axon‐shaped structures). In contrast, in the cerebellum, tdT^+^ was observed in the blood vessels (empty white arrowheads) and Purkinje neurons (empty white arrows), a class of GABAergic inhibitory neurons whose cell bodies showed positive staining for GFAP (Fig. 7‐9*C‐h*).

As we observed tdT expressed in vessel/neural axon‐shaped structures in the brain [Data S7‐1 and S7‐2 (3D view video)], we applied immunofluorescence staining with antibodies against EMCN, tyrosine hydroxylase (TH), or peripherin to identify these tdT^+^ structures. However, the results were not convincing and are not shown here. Further investigation is required. Nevertheless, we observed EMCN expression in tdT^+^ veins of the falx cerebri (Data S8).

### Validation of Ctsk‐Cre expression

Finally, we further verified the expression profile described previously by droplet digital RT‐PCR.

It was recently established that dd‐RT‐PCR is a sensitive and suitable method for the absolute quantification of genes of interest, with the advantages of direct measurement by endpoint, eliminating the need for a calibration standard curve, as required in quantitative real‐time RT‐PCR.^(^ ^29^, ^35^, ^36^, ^37^ ^)^ Therefore, we applied droplet digital RT‐PCR to validate the Ctsk expression profile using mRNA obtained from 10‐week‐old B6 mice.

**Fig. 8:**
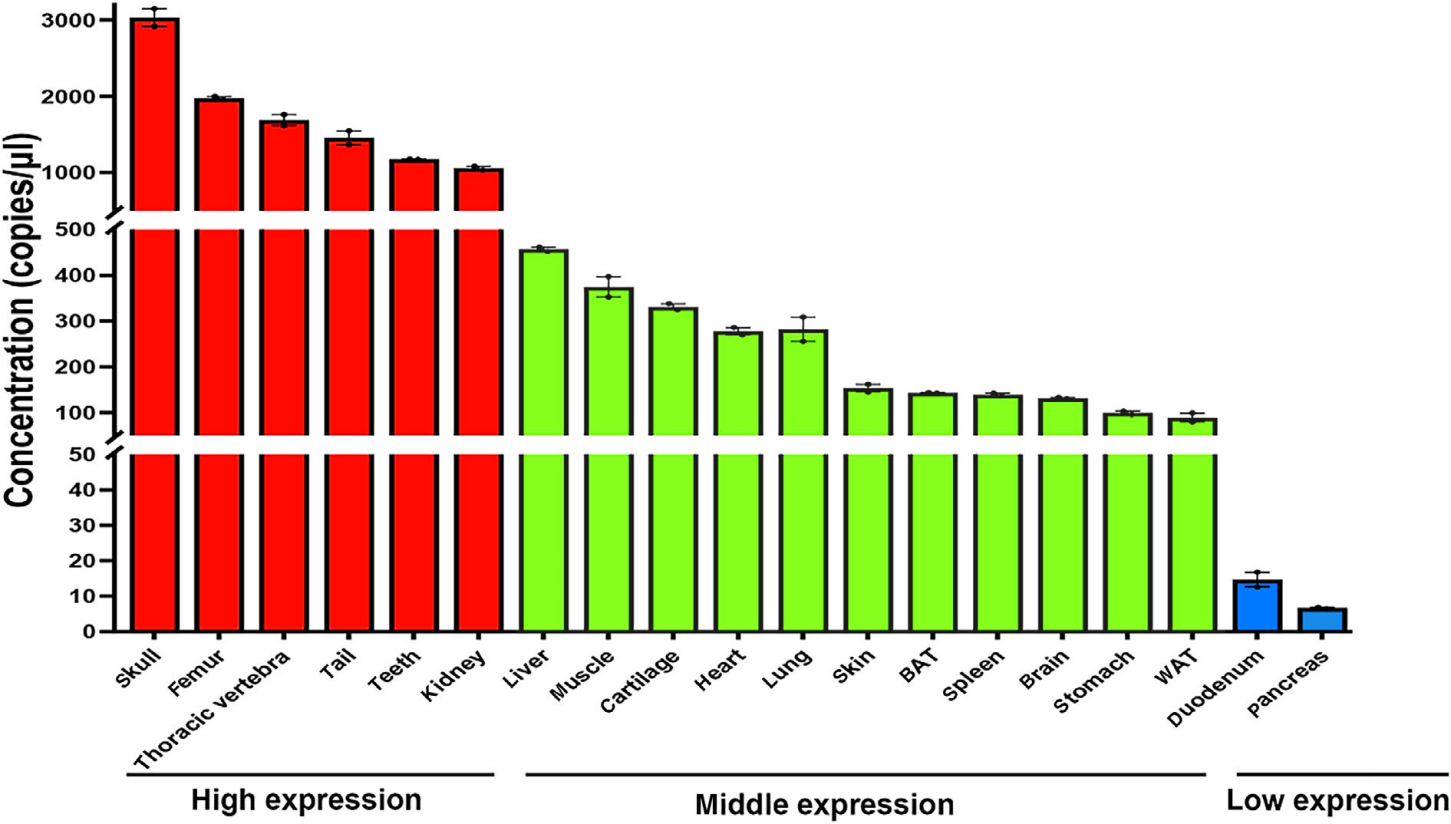
Validation of Ctsk expression in tissues and organs of B6 mice by dd‐RT–PCR. The expression profiles of Ctsk described above were validated by dd‐RT–PCR. dd‐RT–PCR was performed as described in Methods using mRNA from 2‐month‐old B6 mice (male). The expression data were categorized into three groups: high, middle, and low.

As shown in Fig. 8, we detected the highest expression of Ctsk in the skeletal tissues (femur, thoracic spine, and teeth) and the kidney, with copy numbers ranging from 1000 to 2000 copies/μL; intermediate expression in the liver, skeletal muscle, cartilage, heart, lung, skin, BAT, WAT, spleen, brain, and stomach, ranging from 100 to 500 copies/μL; and the lowest expression in the pancreas and duodenum, ranging from 5 to 10 copies/μL.

Ctsk‐Cre expression in the examined tissues/organs is summarized in Table 2.

**Table 2:** Ctsk‐Cre Expression in Examined Transgenic Reporter Mice

Types of tissues/organs	Locations	Cell types
Bone	Bone	Osteoblasts, osteocytes, bone lining cells; osteoclasts
	Growth plate	Chondrocytes in aging mice
	Articular cartilage	Chondrocytes in aging mice
	Bone marrow	Mononuclear cells and osteoclasts; endothelial cells
	Periosteum	Skeletal progenitor cells
	Calvaria	Skeletal progenitor cells, osteoblasts, osteocytes, bone lining cells; osteoclasts
	IVD	Cells in nucleus pulposus and annulus fibrosus
Other tissues/organs	BAT	Brown and endothelial cells, large nerve fibers
	WAT	Beige and endothelial cells, large nerve fibers
	Kidney	Cells in glomerulus, renal tubes; adrenal gland
	Liver	Hepatocytes, cells in portal venule and hepatic artery
	Lung	Bronchiole: Clara secretory cells/goblet cells, mucus‐producing cells; basal cells; neuroendocrine cell/putative early progenitors; smooth muscle in arteriole; type II alveolar cells/macrophages
	Muscle	Cells in endomysium and perimysium, muscle fibers, and satellite cells
	Pancreas	Cells in islets, acinar cells, smooth muscle of arterioles
	Heart	Cardiac muscle fibers
	Testis	Interstitial tissue (IC of Leydig cells); developing spermatozoa (SPZ); seminiferous tubules (ST) [spermatogonia (SG), myoid cells (M), Sertoli cells (SC), late spermatids (LS)]
	Brain	Cells in meninges and dural venous sinuses. Nuclei of pyramidal cells in hippocampus; nuclei of Purkinje neurons in cerebellum, possibly in axons

## Discussion

In this study, we systematically assessed Ctsk‐Cre expression in young and aging Ctsk‐Cre^+^:tdT and Ctsk‐Cre^+^:nT‐nG reporter mice. Our in vitro *and* in vivo results provide evidence at the single‐cell level that Ctsk‐Cre is expressed not only in osteoclasts but also in skeletal progenitors, mature osteoblasts, bone lining cells, and osteocytes; it is also widely expressed in various cell types of major organs and tissues.

We also found that the expression patterns of Ctsk‐Cre^+^:nT‐nG and Ctsk‐Cre^+^:tdT mice varied. We noticed a difference in tdT and nGFP expression in aging skeletal muscle, with an increase in rdT and a decrease in nGFP expression. Different expression patterns for tdT and nGFP were also found in the IVD with aging, with an increase in tdT but a reduction in nGFP expression. Therefore, more than one reporter line is required to examine the expression of a Cre line, particularly in aging mice. These differences in expression patterns may result from the locations of and distance between loxP sites and the probabilistic nature of recombination.^(^ ^38^, ^39^ ^)^ In this study, tdT displayed clearer expression patterns than nT‐nG in the examined tissues and organs due to its brighter expression.

Although Ctsk has been recently used as an osteoclast marker, in this study, we demonstrated its expression in osteoblast lineage cells. From this study and Debnath et al.,^(^ ^17^ ^)^ it was determined that Ctsk‐Cre was expressed in periosteal stem cells/progenitors and a fraction of bone marrow mesenchymal stem cells (CFU‐Obs). Since our results reveal strong Ctsk expression in both osteoclast and osteoblast lineages, genetic modification of genes of interest with Ctsk‐Cre is expected to trigger changes in both bone formation and bone resorption mediated by osteoclasts and osteoblasts, respectively. Therefore, it is necessary to examine both bone formation and bone resorption to elucidate direct or indirect effects and to be cautious when interpreting in vivo data when Ctsk‐Cre is utilized.

Beyond its expression in bone, it is also essential to consider the widespread expression of Ctsk in other organs and tissues when Ctsk‐Cre lines are applied to investigate bone metabolism. In this work, we used different approaches and detected the strongest expression of Ctsk in skeletal tissues. Intermediate expression was observed in the liver, muscle, cartilage, heart, lung, skin, BAT, WAT, spleen, brain, and stomach, and the lowest expression was observed in the pancreas and duodenum, which is in accordance with the pattern reported in humans.^(^ ^40^, ^41^, ^42^, ^43^, ^44^, ^45^ ^)^ Previously, cathepsin K deficiency was reported to cause structural and metabolic changes in the central nervous system (CNS) associated with learning and memory deficits.^(^ ^46^ ^)^ On the other hand, Ctsk deficiency also alleviated aging‐induced cardiac dysfunction^(^ ^47^, ^48^ ^)^ and reduced adiposity during the rapid accumulation of fat stores.^(^ ^20^, ^49^ ^)^ Altogether, these works also indicate widespread expression of Ctsk in tissues and organs.

We used immunofluorescence with antibodies that recognize markers of each cell type to authenticate the identities of tdT^+^ cells in the examined tissues/organs. Unexpectedly, macrophages in several tissues were not tdT‐positive cells, although tdT is expressed in osteoclasts, which share stem/progenitor cells with macrophages. These tissues include the liver, WAT, pancreas, and brain. We also observed that tdT was expressed not only in skeletal muscle (muscle fibers and satellites) but also in smooth muscle in the artery, indicating a role of Ctsk in muscles and blood vessels; an increased serum level of CTSK was also observed in patients with coronary artery disease^(^ ^50^ ^)^ and is involved in muscle degeneration and regeneration.^(^ ^51^ ^)^ In addition, we defined two types of neural cells expressing tdT: pyramidal cells in the hippocampus and Purkinje neurons in the cerebellum. tdT expression in the brain also implies its multiple roles in the CNS. It has been reported that Ctsk‐deficient male mice displayed a decrease in hippocampal astrocytes and altered neuronal patterning and learning and memory deficits,^(^ ^52^ ^)^ supporting our observations in the CNS. In addition, Ctsk expression in the testis observed in this study and in Winkeler et al.^(^ ^21^ ^)^ explains its widespread expression in tissues/organs/cells. Therefore, our findings strongly indicate that suitable controls should be cautiously selected when this Cre line is used for phenotyping knockouts and transgenic mice.

Because of the widespread expression of Ctsk‐Cre in cells other than osteoclasts, it is essential to develop dual promoter‐mediated Cre systems for more precise genetic targeting to osteoclasts, for example, split‐Cre^(^ ^53^, ^54^, ^55^, ^56^ ^)^ and Dre‐Cre.^(^ ^57^, ^58^, ^59^ ^)^ Recent progress in bulk sequencing and scRNA‐seq will provide an opportunity to choose the optimal combination of two promoters to allow Cre to be more accurately expressed in osteoclasts for genetic manipulation.

## Author Contributions

**Wenhuan Chai:** Data curation; formal analysis; investigation; methodology; validation. **Weiwei Hao:** Formal analysis. **Jintao Liu:** Methodology; validation. **Zhenglin Han:** Data curation. **Shiyu Chang:** Data curation. **Liben Cheng:** Methodology. **Mingxin Sun:** Data curation. **Guofang Yan:** Resources. **Zemin Liu:** Validation; visualization. **Yin Liu:** Resources. **Guodong Zhang:** Visualization. **Li Xing:** Resources. **Hongqian Chen:** Data curation; formal analysis; investigation; project administration; resources; supervision; validation; visualization. **Peng Liu:** Conceptualization; formal analysis; funding acquisition; investigation; supervision; validation; writing – original draft; writing – review and editing.

### Peer Review

The peer review history for this article is available at https://publons.com/publon/10.1002/jbm4.10706.

## Supporting information


**Data S1.** Authentication of Ctsk‐Cre mice by Ctsk‐Cre site‐specific genotyping PCR. The obtained Ctsk‐Cre line was authenticated by site‐specific genotyping PCR as described previously, with an amplicon size of 992 bp, as indicated.^25^ N: negative control without Cre; B: blank control (ddH_2_O); M: molecular DNA marker (2 kb).

**Data S2.** Negative controls for imaging nT‐nG and tdT in absence of Ctsk‐Cre. Various tissues (BAT, WAT, brain, kidney, liver, lung, muscle, pancreas, trabecular bone, IVD, calvaria, and cartilage) from nT‐nG and tdT reporter lines were imaged as negative controls for the expression of either nT‐nG (*A*) or tdT (*B*) crossed with Ctsk‐Cre mice. Images were taken under the same conditions used for imaging positive expression in each tissue/organ. Magnification: ×400. Scale bar: 50 μm.

**Data S3.** Calcitonin R expression in osteocytes of patella and EMCN expression in epiphysis. Immunofluorescence staining showed calcitonin R expression in cortical bone of patella (*A*) and EMCN expression in tdT^−^ type H vessels indicated by red empty arrows (*B*); interestingly, tdT^−^ chondrocytes in the growth plate were enveloped by EMCN‐stained vessels, indicated by white empty arrows. Arrows in a: white: osteocytes with both tdT and calcitonin R expression; empty white: osteocytes with only calcitonin R expression. Arrows in b: empty red: type H vessels; empty white: chondrocytes circumscribed by EMCN positively stained vessels. Scale bar: 20 μm. Magnification: ×1000.

**Data S4.** Defining expression of TH in tdT^+^ BAT tissue. To determine whether TH is expressed in tdT^+^ peripheral never fiber‐shaped structures, we performed immunofluorescence staining with an anti‐TH antibody in frozen BAT sections. In large peripheral nerve fibers (fascicle), TH was seen in the neurofibrils/axons indicated by blue arrows, which were surrounded by a tdT‐positive epineural sheath (epineurium) indicated by white arrows (*A* and *B*). However, small never fibers were found to partially colocalize with tdT^+^ fiber structures (*B* and *C*). Interestingly, we also found that TH was not only expressed in the never fibers but was also highly expressed in tdT^+^ browning cells. In (*A*) (detection of TH expression in large never fibers): (*a*) TH expression in BAT at magnification of ×400; (*b*) 3D view of never TH‐positive fibrils. In (*B*) (confirmation of TH expression of TH in large nerve fibrils), (*a*) further confirmation of TH expression at magnification of ×400, (*b, c*) different 3D section views of large never fibrils at magnification of ×1000. In (*C*) (partial colocalization of TH and tdT in small never fibrils), (*a*) TH expression at magnification of ×400; (*b, c*) different 3D section views of TH and tdT expression at magnification of ×1000. Arrows: white: large never fibrils; blue: positively stained neurofibrils/axons; empty white: TH positively stained small fibers colocalized with tdT fiber structures; white arrowheads: TH positively stained brown cells; empty white arrowheads: brown cells with both tdT and TH expression. Scale bars: 50 μm for A and B; 20 μm for (*C*). Magnifications: ×400 for (*A*) and (*B*), and ×1000 for (*C*).

**Data S5.** TH expression in brown cells. Interestingly, TH expression was observed in tdT^+^ brown cells shown at magnifications of ×400 (*A*) and ×1000 (*B*). Arrowheads: white: brown cells stained positively only for TH; empty white: brown cells with both tdT expression and Th staining. Scale bars: 50 μm for (*A*) and 20 μm for (*B*) Magnifications: ×400 for (*A*) and ×1000 for (*B*).

**Data S6.** Immunofluorescence images of brain sections with NeuN antibody. A NeuN antibody was utilized for tdT^+^ cell identification in horizontal brain sections of Ctsk‐Cre^+^:tdT mice. The imaged areas are as follows: (*A*) olfactory bulb (×40). Arrows: white arrow: neural dendrites; empty white arrowhead: cilia layer. Layers: 1: glomerular layer; 2: external plexiform layer; 3: mitral cell layer; 4: internal plexiform layer; 5: granule cell layer; 6: anterior commissure. (*B*) Cerebral cortex (×200). Arrows: white blood vessels and empty white arrowhead: neural dendrites. Layers: I: molecular layer (plexiform layer); II: external granular layer; III: external pyramidal cell layer; IV: internal granular layer; V: internal pyramidal cell layer; VI: polymorphic layer (multiform layer). (*C*) Cerebral cortex (III, IV, and V) (×400). Arrows: white vascellum; empty white arrowhead: body of pyramidal cells; yellow arrow: small granular cells; small white arrows: axon of pyramidal cells. Layers: III, IV, and V. (*D*) Cerebral cortex (V) (×400). Arrows: white: pyramidal cells; empty white arrowhead: vascellum. (*E*) Thalamus (×400). Arrows: white neurons; empty arrowhead: vascellum. (*F*) Hypothalamus (LH, lateral hypothalamus, ×400). Arrows: white: vessel; empty white arrowheads: neurons positive for NeuN. (*G*) Medulla (×200). Arrows: artery; empty white arrows: motor neurons; empty white arrowheads: vascellum.

**Data S7‐1.** tdT expression in vessel/neural axon‐shaped structures in the cerebral cortex. tdT^+^ vessel/neural axon‐shaped structures in the cerebral cortex were imaged at a magnification of 400X. Scale bar: 50 μm.

**Data S7‐2.** 3D view of tdT expression in axon‐shaped structures in cerebral cortex.

**Data S8.** EMCN expression in tdT^+^ veins of falx cerebri. EMCN was positively stained in tdT^+^ veins of falx cerebri. Scale bar: 50. Magnification: ×400.

**Appendix S1.** Supplemental Material. Schematic map for anatomic locations of neuroimages in horizontal brain sections, referenced by Allen adult mouse brain atlas.

## Data Availability

The data that support the findings of this study are available in the supplementary material of this article. Source data for all experiments are available upon reasonable request.
