# Supplementary figures and images for: Visualizing Cathepsin K‐Cre Expression at the Single‐Cell Level with GFP Reporters
Source: JBMR Plus. 2022 Dec 21;7(1):e10706. doi: 10.1002/jbm4.10706 (PMC9850439; doi:10.1002/jbm4.10706)

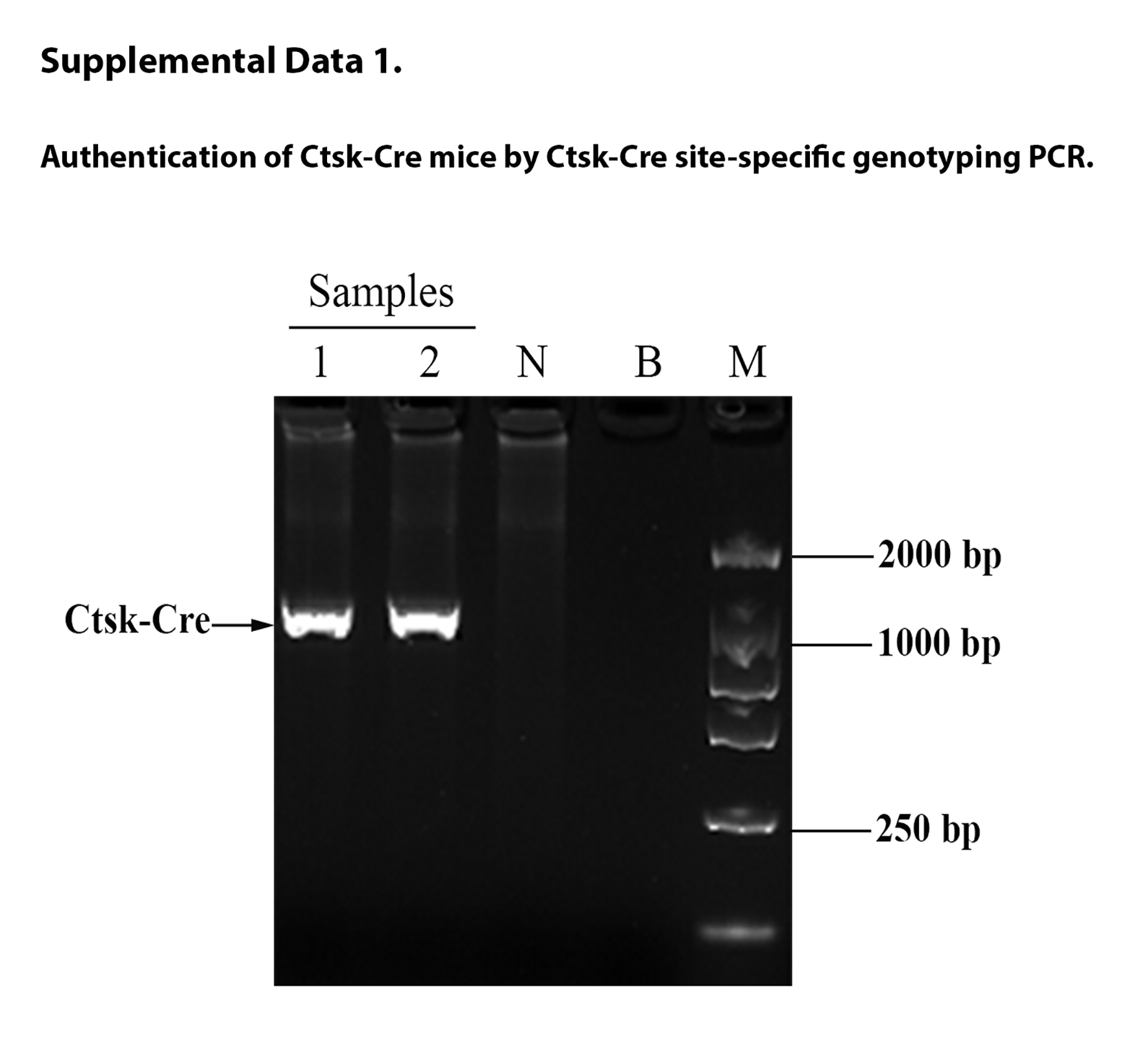

Supplement: Supplementary file 1 — Data S1. Authentication of Ctsk‐Cre mice by Ctsk‐Cre site‐specific genotyping PCR. The obtained Ctsk‐Cre line was authenticated by site‐specific genotyping PCR as described previously, with an amplicon size of 992 bp, as indicated.25 N: negative control without Cre; B: blank control (ddH2O); M: molecular DNA marker (2 kb). [file JBM4-7-e10706-s008.tif]

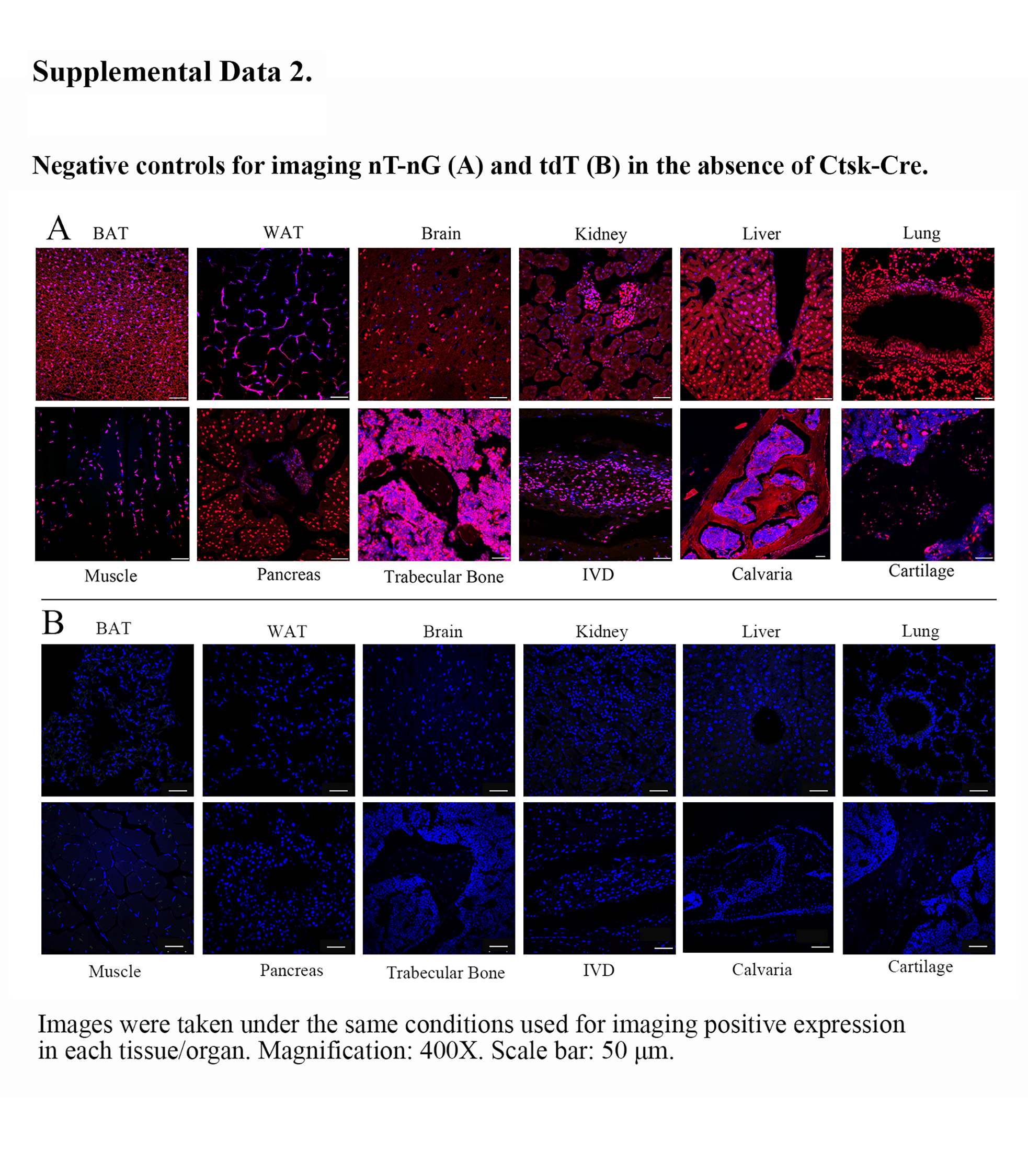

Supplement: Supplementary file 2 — Data S2. Negative controls for imaging nT‐nG and tdT in absence of Ctsk‐Cre. Various tissues (BAT, WAT, brain, kidney, liver, lung, muscle, pancreas, trabecular bone, IVD, calvaria, and cartilage) from nT‐nG and tdT reporter lines were imaged as negative controls for the expression of either nT‐nG (A) or tdT (B) crossed with Ctsk‐Cre mice. Images were taken under the same conditions used for imaging positive expression in each tissue/organ. Magnification: ×400. Scale bar: 50 μm. [file JBM4-7-e10706-s001.tif]

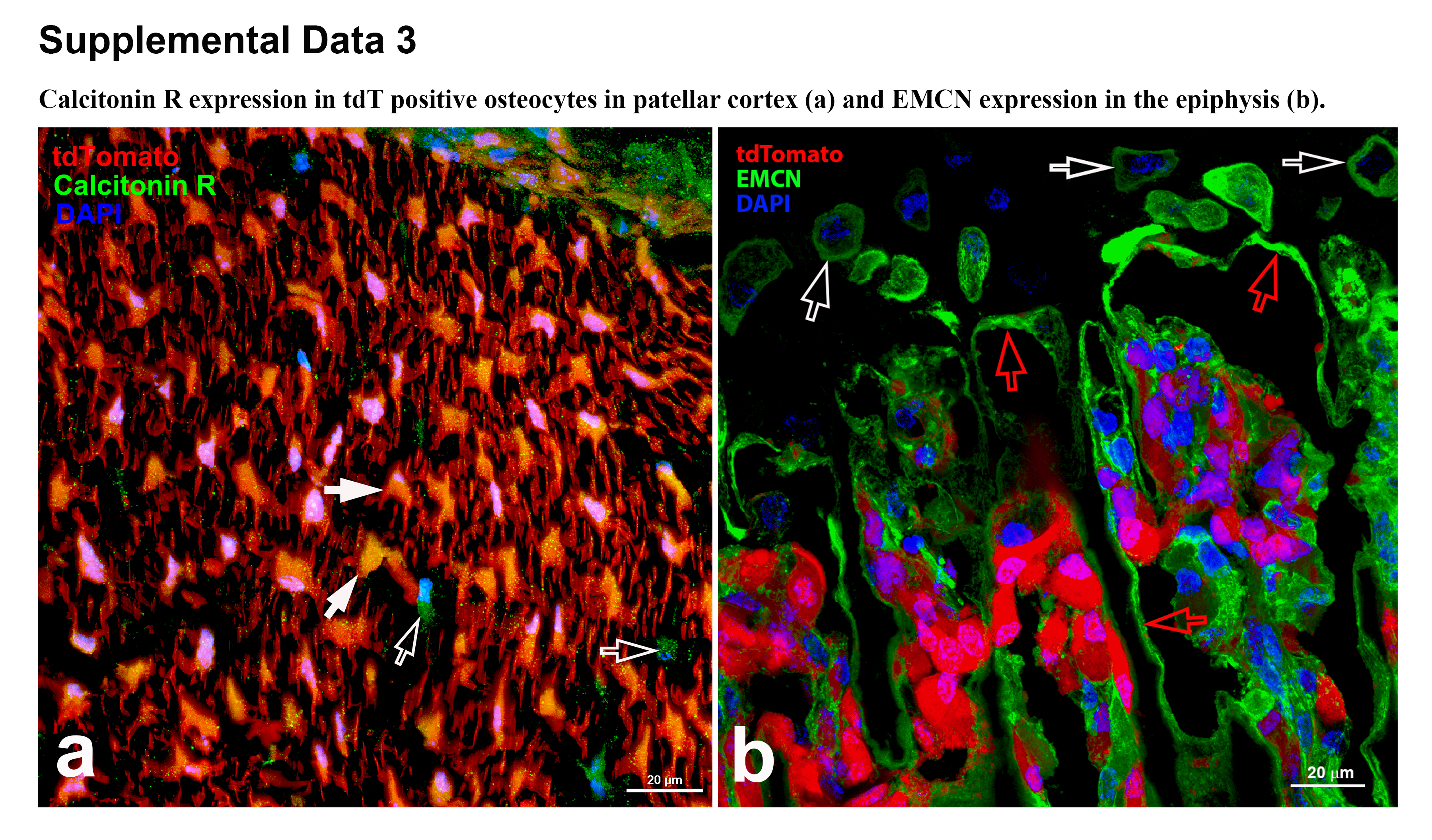

Supplement: Supplementary file 3 — Data S3. Calcitonin R expression in osteocytes of patella and EMCN expression in epiphysis. Immunofluorescence staining showed calcitonin R expression in cortical bone of patella (A) and EMCN expression in tdT− type H vessels indicated by red empty arrows (B); interestingly, tdT− chondrocytes in the growth plate were enveloped by EMCN‐stained vessels, indicated by white empty arrows. Arrows in a: white: osteocytes with both tdT and calcitonin R expression; empty white: osteocytes with only calcitonin R expression. Arrows in b: empty red: type H vessels; empty white: chondrocytes circumscribed by EMCN positively stained vessels. Scale bar: 20 μm. Magnification: ×1000. [file JBM4-7-e10706-s007.tif]

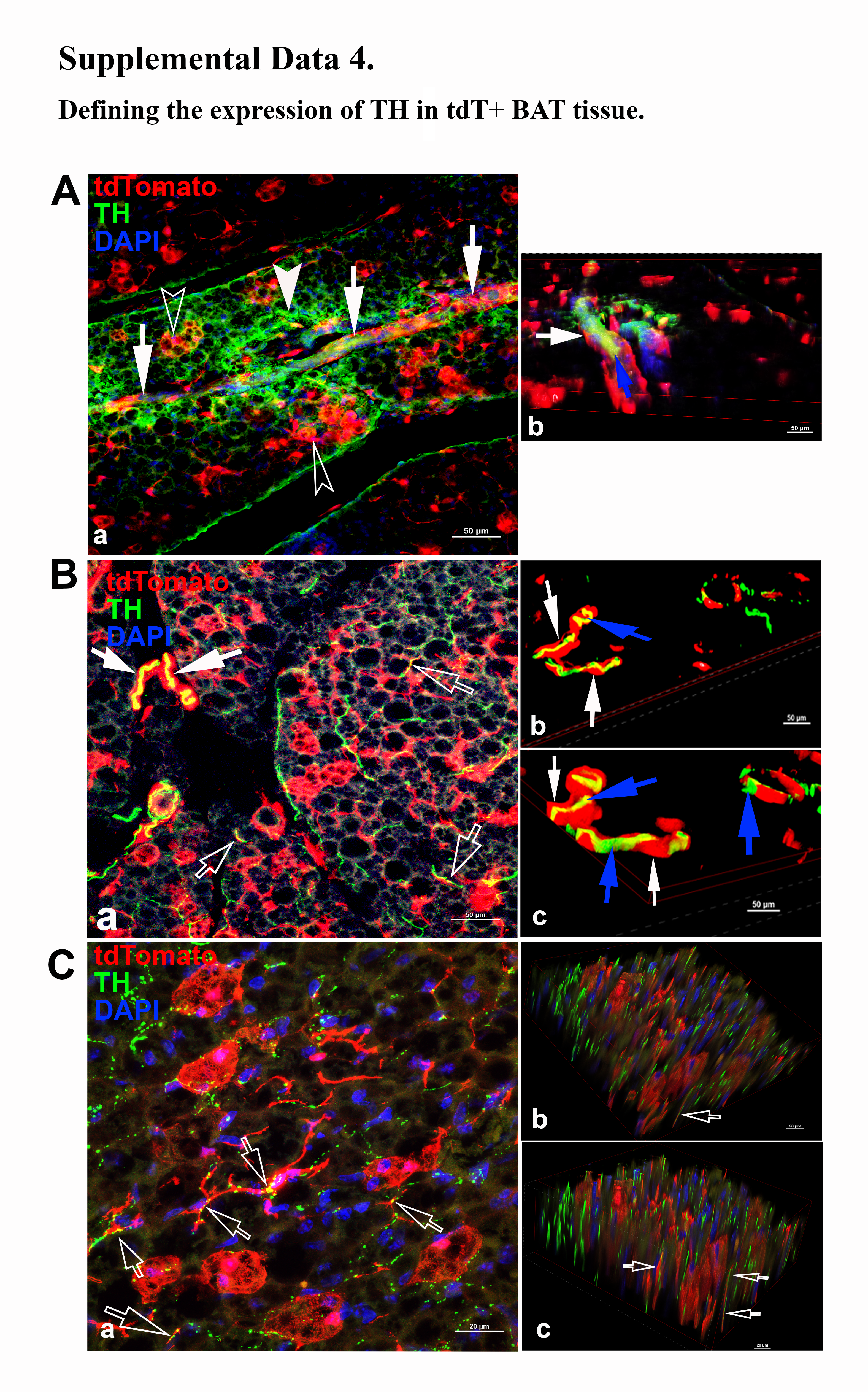

Supplement: Supplementary file 4 — Data S4. Defining expression of TH in tdT+ BAT tissue. To determine whether TH is expressed in tdT+ peripheral never fiber‐shaped structures, we performed immunofluorescence staining with an anti‐TH antibody in frozen BAT sections. In large peripheral nerve fibers (fascicle), TH was seen in the neurofibrils/axons indicated by blue arrows, which were surrounded by a tdT‐positive epineural sheath (epineurium) indicated by white arrows (A and B). However, small never fibers were found to partially colocalize with tdT+ fiber structures (B and C). Interestingly, we also found that TH was not only expressed in the never fibers but was also highly expressed in tdT+ browning cells. In (A) (detection of TH expression in large never fibers): (a) TH expression in BAT at magnification of ×400; (b) 3D view of never TH‐positive fibrils. In (B) (confirmation of TH expression of TH in large nerve fibrils), (a) further confirmation of TH expression at magnification of ×400, (b, c) different 3D section views of large never fibrils at magnification of ×1000. In (C) (partial colocalization of TH and tdT in small never fibrils), (a) TH expression at magnification of ×400; (b, c) different 3D section views of TH and tdT expression at magnification of ×1000. Arrows: white: large never fibrils; blue: positively stained neurofibrils/axons; empty white: TH positively stained small fibers colocalized with tdT fiber structures; white arrowheads: TH positively stained brown cells; empty white arrowheads: brown cells with both tdT and TH expression. Scale bars: 50 μm for A and B; 20 μm for (C). Magnifications: ×400 for (A) and (B), and ×1000 for (C). [file JBM4-7-e10706-s002.tif]

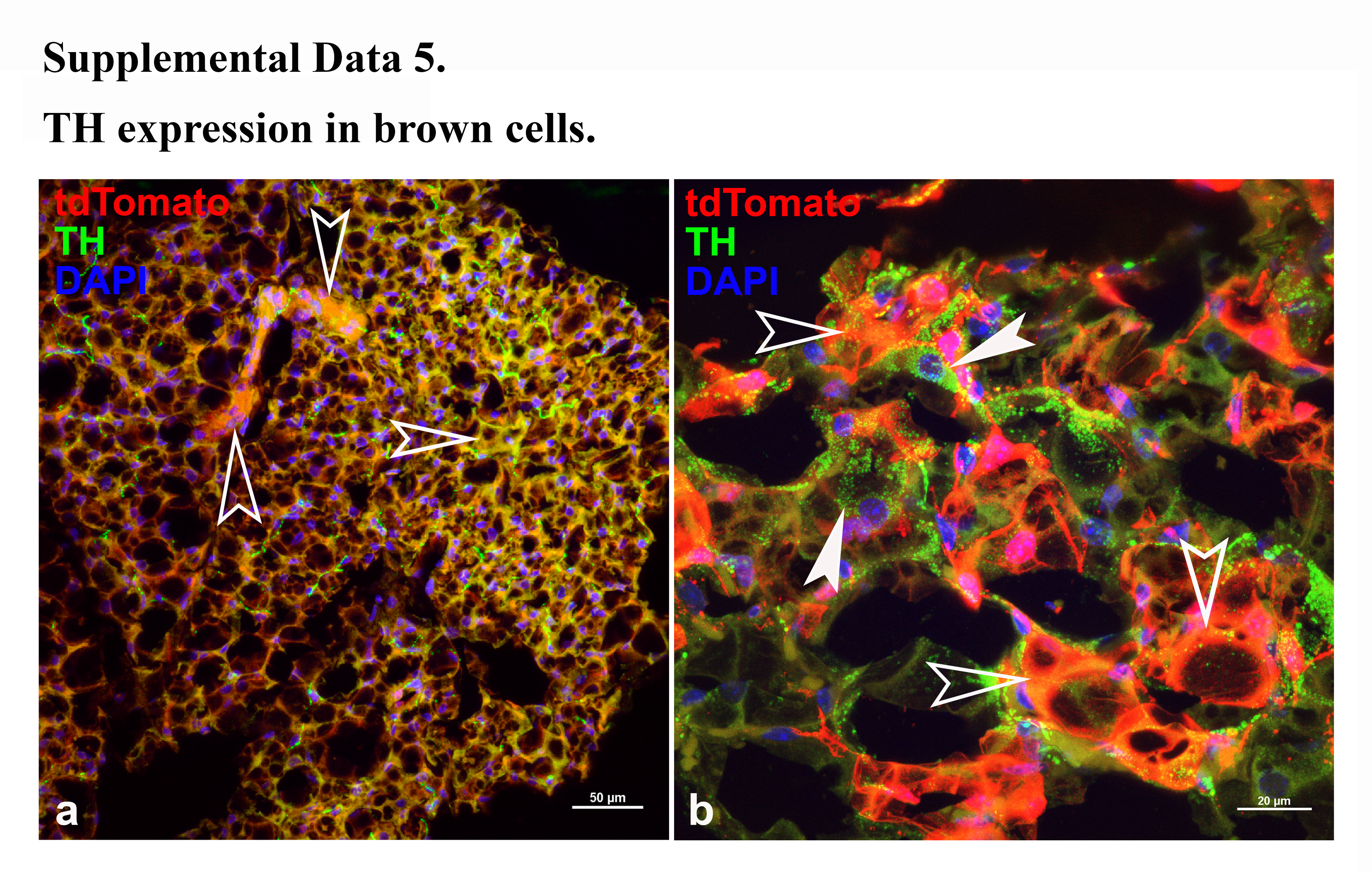

Supplement: Supplementary file 5 — Data S5. TH expression in brown cells. Interestingly, TH expression was observed in tdT+ brown cells shown at magnifications of ×400 (A) and ×1000 (B). Arrowheads: white: brown cells stained positively only for TH; empty white: brown cells with both tdT expression and Th staining. Scale bars: 50 μm for (A) and 20 μm for (B) Magnifications: ×400 for (A) and ×1000 for (B). [file JBM4-7-e10706-s005.tif]

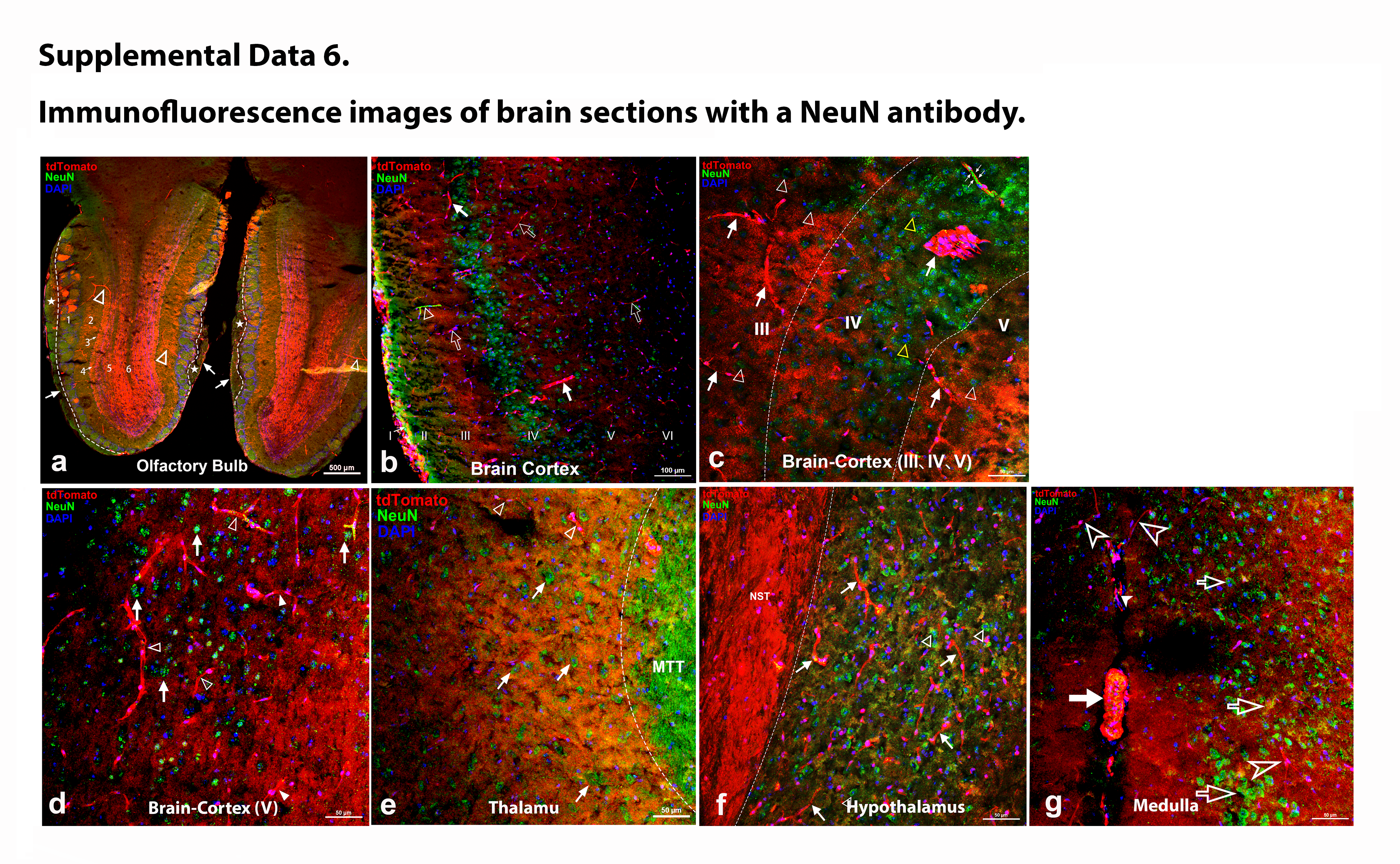

Supplement: Supplementary file 6 — Data S6. Immunofluorescence images of brain sections with NeuN antibody. A NeuN antibody was utilized for tdT+ cell identification in horizontal brain sections of Ctsk‐Cre+:tdT mice. The imaged areas are as follows: (A) olfactory bulb (×40). Arrows: white arrow: neural dendrites; empty white arrowhead: cilia layer. Layers: 1: glomerular layer; 2: external plexiform layer; 3: mitral cell layer; 4: internal plexiform layer; 5: granule cell layer; 6: anterior commissure. (B) Cerebral cortex (×200). Arrows: white blood vessels and empty white arrowhead: neural dendrites. Layers: I: molecular layer (plexiform layer); II: external granular layer; III: external pyramidal cell layer; IV: internal granular layer; V: internal pyramidal cell layer; VI: polymorphic layer (multiform layer). (C) Cerebral cortex (III, IV, and V) (×400). Arrows: white vascellum; empty white arrowhead: body of pyramidal cells; yellow arrow: small granular cells; small white arrows: axon of pyramidal cells. Layers: III, IV, and V. (D) Cerebral cortex (V) (×400). Arrows: white: pyramidal cells; empty white arrowhead: vascellum. (E) Thalamus (×400). Arrows: white neurons; empty arrowhead: vascellum. (F) Hypothalamus (LH, lateral hypothalamus, ×400). Arrows: white: vessel; empty white arrowheads: neurons positive for NeuN. (G) Medulla (×200). Arrows: artery; empty white arrows: motor neurons; empty white arrowheads: vascellum. [file JBM4-7-e10706-s004.tif]

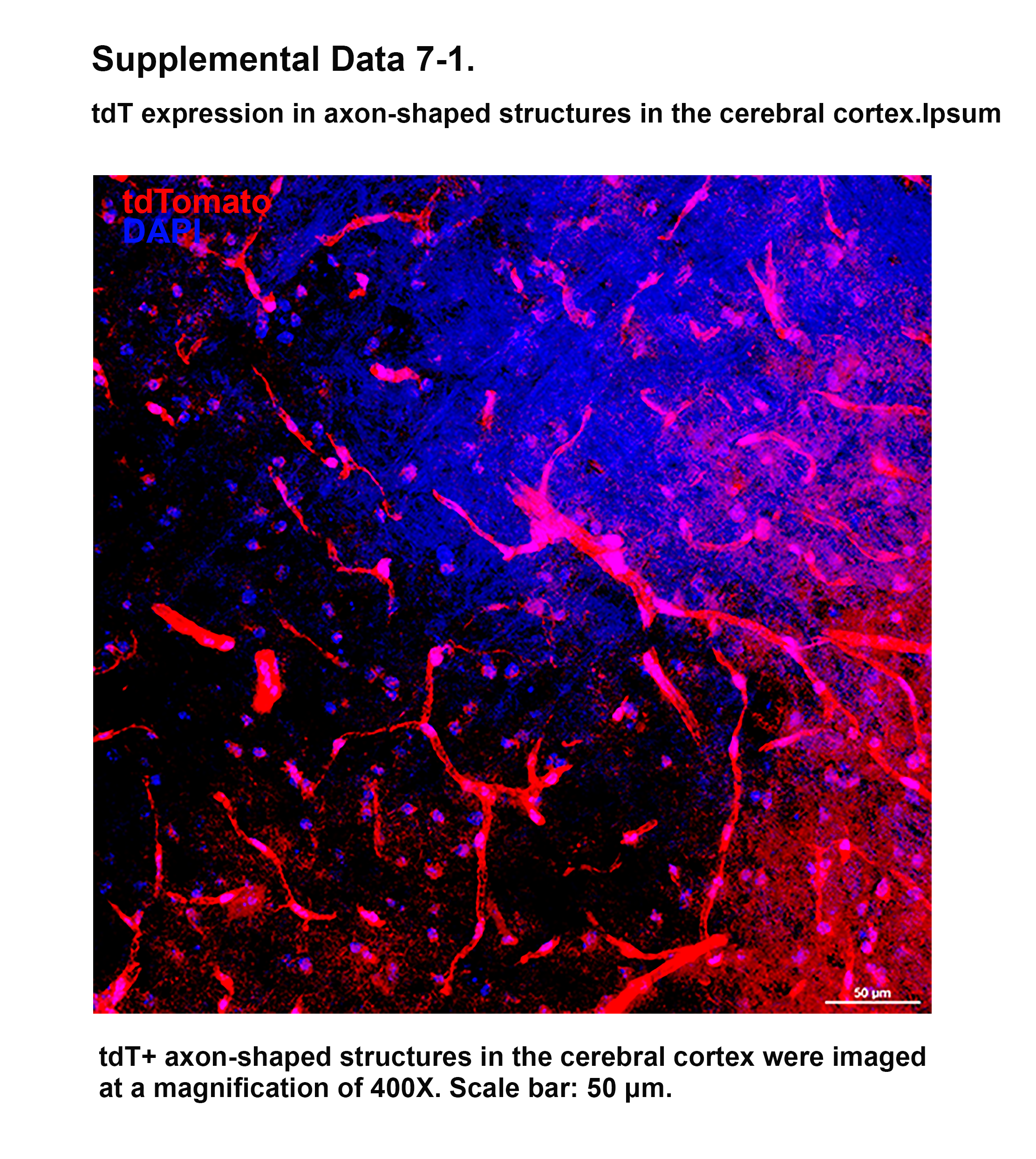

Supplement: Supplementary file 7 — Data S7‐1. tdT expression in vessel/neural axon‐shaped structures in the cerebral cortex. tdT+ vessel/neural axon‐shaped structures in the cerebral cortex were imaged at a magnification of 400X. Scale bar: 50 μm. [file JBM4-7-e10706-s006.tif]

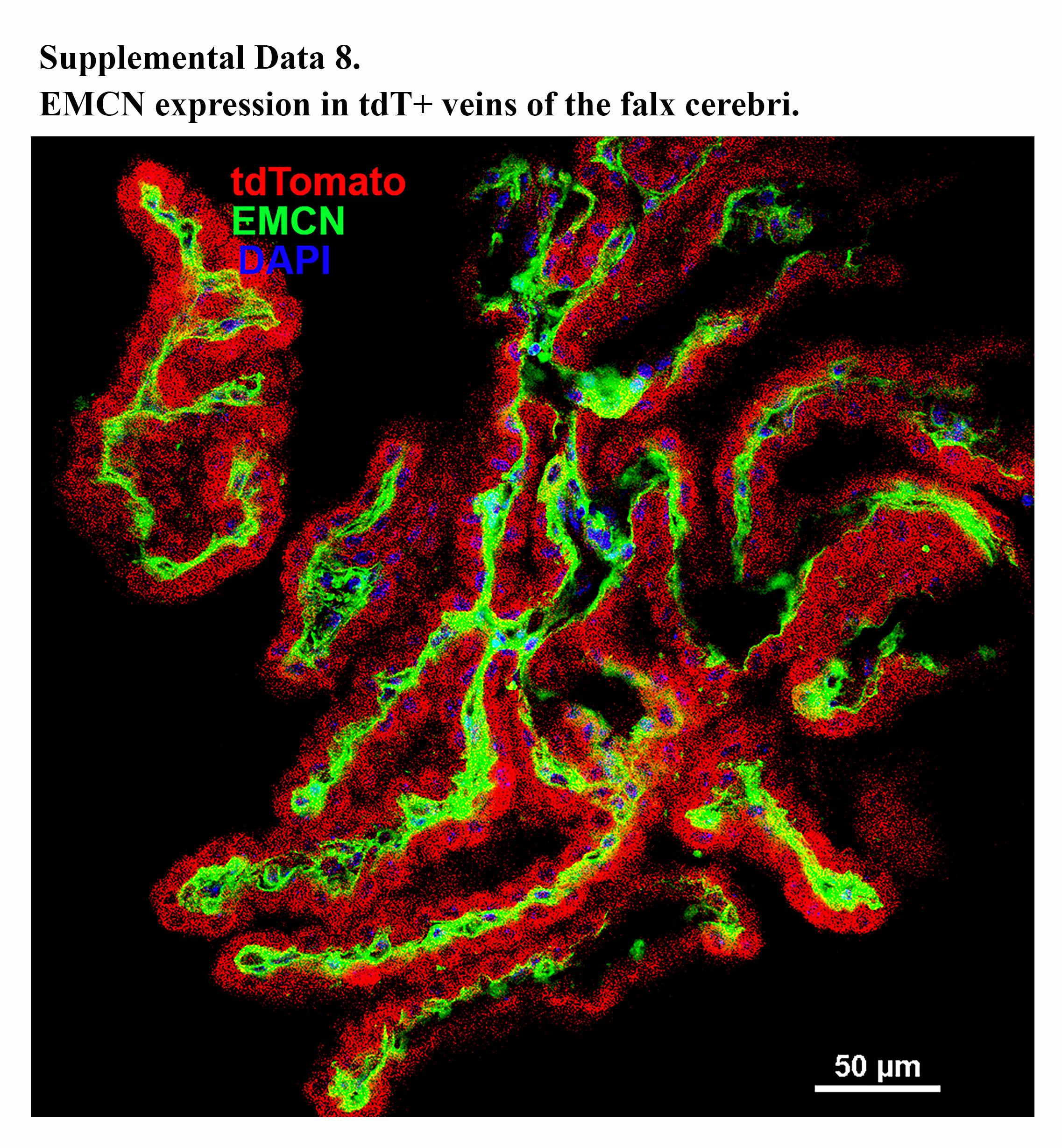

Supplement: Supplementary file 9 — Data S8. EMCN expression in tdT+ veins of falx cerebri. EMCN was positively stained in tdT+ veins of falx cerebri. Scale bar: 50. Magnification: ×400. [file JBM4-7-e10706-s010.tif]
