# Supplementary material for: Visualizing Cathepsin K‐Cre Expression at the Single‐Cell Level with GFP Reporters
Source: JBMR Plus. 2022 Dec 21;7(1):e10706. doi: 10.1002/jbm4.10706 (PMC9850439; doi:10.1002/jbm4.10706)
Supplement: Supplementary file 10 — Appendix S1. Supplemental Material. Schematic map for anatomic locations of neuroimages in horizontal brain sections, referenced by Allen adult mouse brain atlas. [file JBM4-7-e10706-s009.pdf]

Supplemental Material

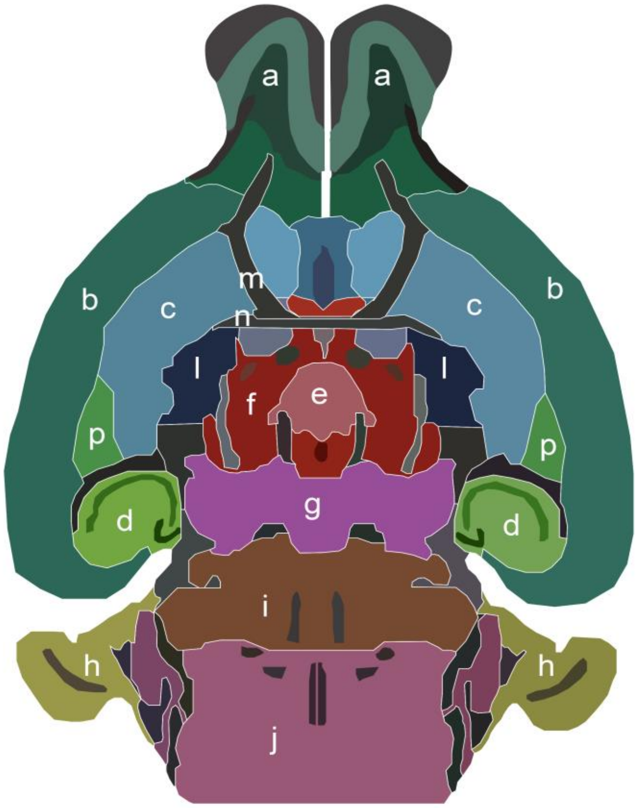


**A schematic map for anatomic locations of the mouse brain in horizontal sections referenced by the Allen adult mouse brain atlas**

**a**-Olfactory bulb

**b**-Cerebral cortex

**c**-Striatum

**d**-Hippocampal

**e**-Thalamus

**f**-Hypothalamus

**g**-Midbrain

**h**-Cerebellum

**i**-Pons

**j**-Medulla,

**k**-Corticospinal tract

**l**-Pallidum

**m**-Anterior commissure olfactory limb

**n**-Corpus callosum

**o**-Lateral ventricles
